# A clinically attenuated double-mutant of porcine reproductive and respiratory syndrome virus-2 that does not prompt overexpression of proinflammatory cytokines during co-infection with a secondary pathogen

**DOI:** 10.1371/journal.ppat.1012128

**Published:** 2024-03-28

**Authors:** Chia-Ming Su, Jineui Kim, Junyu Tang, Yu Fan Hung, Federico A. Zuckermann, Robert Husmann, Patrick Roady, Jiyoun Kim, Young-Min Lee, Dongwan Yoo

**Affiliations:** 1 Department of Pathobiology, College of Veterinary Medicine, University of Illinois at Urbana-Champaign, Urbana, Illinois, United States of America; 2 Department of Veterinary Clinical Medicine, College of Veterinary Medicine, University of Illinois at Urbana-Champaign, Urbana, Illinois, United States of America; 3 Department of Animal, Dairy, and Veterinary Sciences, Utah State University, Logan, Utah, United States of America; Leiden University Medical Center, NETHERLANDS

## Abstract

Porcine reproductive and respiratory syndrome virus (PRRSV) is known to suppress the type I interferon (IFNs-α/β) response during infection. PRRSV also activates the NF-κB signaling pathway, leading to the production of proinflammatory cytokines during infection. In swine farms, co-infections of PRRSV and other secondary bacterial pathogens are common and exacerbate the production of proinflammatory cytokines, contributing to the porcine respiratory disease complex (PRDC) which is clinically a severe disease. Previous studies identified the non-structural protein 1β (nsp1β) of PRRSV-2 as an IFN antagonist and the nucleocapsid (N) protein as the NF-κB activator. Further studies showed the leucine at position 126 (L126) of nsp1β as the essential residue for IFN suppression and the region spanning the nuclear localization signal (NLS) of N as the NF-κB activation domain. In the present study, we generated a double-mutant PRRSV-2 that contained the L126A mutation in the nsp1β gene and the NLS mutation (ΔNLS) in the N gene using reverse genetics. The immunological phenotype of this mutant PRRSV-2 was examined in porcine alveolar macrophages (PAMs) in vitro and in young pigs in vivo. In PAMs, the double-mutant virus did not suppress IFN-β expression but decreased the NF-κB-dependent inflammatory cytokine productions compared to those for wild-type PRRSV-2. Co-infection of PAMs with the mutant PRRSV-2 and *Streptococcus suis (S*. *suis)* also reduced the production of NF-κB-directed inflammatory cytokines. To further examine the cytokine profiles and the disease severity by the mutant virus in natural host animals, 6 groups of pigs, 7 animals per group, were used for co-infection with the mutant PRRSV-2 and *S*. *suis*. The double-mutant PRRSV-2 was clinically attenuated, and the expressions of proinflammatory cytokines and chemokines were significantly reduced in pigs after bacterial co-infection. Compared to the wild-type PRRSV-2 and *S*. *suis* co-infection control, pigs coinfected with the double-mutant PRRSV-2 exhibited milder clinical signs, lower titers and shorter duration of viremia, and lower expression of proinflammatory cytokines. In conclusion, our study demonstrates that genetic modification of the type I IFN suppression and NF-κB activation functions of PRRSV-2 may allow us to design a novel vaccine candidate to alleviate the clinical severity of PRRS-2 and PRDC during bacterial co-infection.

## Author summary

Porcine reproductive and respiratory syndrome virus (PRRSV) is a significant swine pathogen worldwide. PRRSV suppresses the type I interferon (IFN) response and activates the NF-κB signaling, leading to the production of proinflammatory cytokines. Co-infection of PRRSV and a secondary pathogen is common in farms and exacerbates the production of proinflammatory cytokines, contributing to the porcine respiratory disease complex (PRDC) which is a clinically severe disease. Here, we generated a double-mutant PRRSV-2 in the nsp1β and N genes by reverse genetics. In cells, the mutant virus did not suppress IFN expression and reduced the NF-κB-mediated inflammatory cytokines compared to wild-type PRRSV-2. Co-infection of macrophages with the mutant virus and *Streptococcus suis* reduced the production of inflammatory cytokines. The immunological phenotypes of the mutant PRRSV-2 were examined in pigs, 6 groups of pigs, 7 animals per group, after co-infection with *S*. *suis*. The double-mutant PRRSV-2 was clinically attenuated, and the expressions of proinflammatory cytokines were significantly reduced in pigs after bacterial co-infection. Our study provides insights that the reprogramming of viral immune antagonism may allow to design of a novel vaccine candidate to alleviate the clinical severity of PRRS and PRDC during co-infection.

## Introduction

The host innate immunity contributes to the first line of defense against viral infections, and type I interferons (IFNs-α/β) are the most potent antiviral cytokines produced in cells against invading viruses. Type I IFNs are considered principle antiviral cytokines, but a large body of evidence indicates that they also play a pleiotropic role in regulating the adaptive immunity. IFNs can enhance the adaptive immunity by targeting dendritic cells, natural killer cells, T cells, and B cells [1]. In virus-infected mice, type I IFNs stimulate CD4^+^ T cells undergoing clonal expansion [2]. T cells primed by type I IFNs also show an increased ability to help B cells to enhance antibody secretions [3]. In other studies, type I IFNs have been shown to upregulate the survival, maturation, cytotoxicity, and clonal expansion of CD8^+^ T cells [4–14]. In addition, type I IFNs promote the differentiation of memory CD8^+^ T cells by affecting the initial clonal expansion during virus infection [8,15,16]. Type I IFNs can also regulate B cell activation, antibody secretion, and isotype switching during viral infections [17–19]. By increasing the level of B-cell survival factors, type I IFNs can promote B-cell survival and activation and enhance autoantibody production [20].

Porcine reproductive and respiratory syndrome (PRRS) is a swine disease, causing significant economic losses in most pork-producing countries worldwide. As the name suggests, the typical clinical signs of PRRS include reproductive failures in pregnant animals and respiratory disease and pneumonia in all age of pigs with more pronounced symptoms in young pigs. Immunologically, the hallmarks of PRRS are represented by unusually poor production of type I IFNs early in infection, accompanied by low and delayed neutralizing antibody response and viral persistence, suggesting that the adaptive response to invading virus is perturbed [21–23]. Conversely, the downregulation of IFN production by PRRSV seems to be an important viral strategy to evade host antiviral defense to facilitate its own replication.

The etiological agent PRRSV belongs to the family *Arteriviridae* of the order *Nidovirales* (http://www.ictv.global/report/arteriviridae). The PRRSV genome is a single-strand positive-sense RNA of ~15 kb in length, and based on their profound genomic differences, the viruses are assigned to two distinct species *Betaarterivirus suid 1* (PRRSV-1; European genotype) and *Betaarterivirus suid 2 (*PRRSV-2; North American genotype) [24]. PRRSV-1 and PRRSV-2 are often collectively referred to as PRRSV in the literature and in the present article. The genome organization of PRRSV-1 and PRRSV-2 resembles those of coronaviruses in the order *Nidovirales*, but the genome length of PRRSV is approximately half of coronaviruses. The 5’ ~12 kb of the genome codes for two large polyproteins, pp1a and pp1ab, of which the latter is produced by the ribosomal frame-shifting mechanism. The two polyproteins are further processed to generate 14 nonstructural proteins (nsps). The remaining ~3 kb of the 3’ proximity of the genome codes for eight structural proteins: envelope (E), glycoprotein (GP) 2, GP3, GP4, GP5, open reading frame (ORF) 5a, membrane (M), and nucleocapsid (N) proteins [25,26].

Of PRRSV-2 proteins reported to downregulate IFN suppression, nsp1β is the most potent IFN antagonist [27–31]. The nsp1β protein blocks the host mRNAs export from the nucleus to the cytoplasm and allows PRRSV-2 to utilize the cellular translational machinery exclusively for viral protein synthesis and thus promotes progeny production [32]. This function has been correlated with nsp1β-mediated IFNs suppression. A specific motif for SAP [Scaffold attachment factor-A/B, Acinus, and Protein inhibitor of activated STAT (signal transducer and activator of transcription)] has been identified in the nsp1β protein with the consensus sequence of _124_-KxLQxxLxxxGL-_135_ within the papain-like proteinase domain [33]. Mutational analyses in the SAP motif revealed that L126A conferred the loss of host mRNA nuclear retention and nsp1β-mediated type I IFNs suppression. A mutant PRRSV-2 containing L126A was generated, and the phenotype of the mutant PRRSV-2 was host mRNA nuclear retention-negative and type I IFN suppression-negative.

In addition to IFN modulation, PRRSV-2 utilizes NF-κB signaling for its own benefit. Contradictory but complementary data are available for PRRSV-mediated NF-κB regulation. In cells expressing nsp1α, nsp1β, nsp2, nsp4, or nsp11, the NF-κB activity was downregulated, and this downregulation was linked to the inhibition of type I IFNs production pathway [34,35]. In contrast, cells expressing the N protein upregulated the NF-κB activity [36,37]. This finding was supported by the demonstration of N protein-mediated expression of proinflammatory cytokines such as interleukin (IL)-1β, IL-6, IL-8, and TNFα. The molecular basis for NF-κB activation by PRRSV-2 N has been determined [37]. N is specifically distributed in the nucleus and the nucleolus in addition to the cytoplasm. N contains the nuclear localization signal (NLS), and its nuclear localization is NLS-dependent through the binding of N to importins-α/β. We have previously identified protein inhibitor of STAT1 (PIAS1) as the cellular partner of N [37]. PIAS is a negative regulator for the JAK-STAT signaling for antiviral protein expressions and functions as a repressor for NF-κB by binding to the NF-κB subunit p65. PRRSV N binding to PIAS1 overlaps the p65 binding domain. The binding of N to PIAS1 results in the release of p65 from PIAS1, leading to the NF-κB activation [37].

PRRSV-1 and PRRSV-2 are predominant pathogens in many commercial operations. PRRSV increases the susceptibility of infected hosts to secondary pathogens. Co-infection with other pathogens is frequent and causes porcine respiratory disease complex (PRDC), resulting in more severe clinical disease [38–40]. Co-infection increases the secretion of proinflammatory cytokines and exacerbates tissue damages and pulmonary infiltration [40]. Cytokine storm-like overproduction of inflammatory cytokines have been reported for PRRS, and the clinical outcome is more severe in the PRDC animals [41–45]. Since PRRSV N protein upregulates NF-κB, co-infection with PRRSV and a secondary bacterial infection can trigger synergistic activation of NF-κB and overproduction of inflammatory cytokines, which is likely the mechanistic basis for severe pathogenesis and higher mortality.

In the present study, we hypothesized that a mutation in the SAP motif of the nsp1β protein would reverse the viral IFN antagonism and could induce better adaptive immune response, while the PIAS1-binding motif-mutation in N would reserve the NF-κB activation and attenuate the production of proinflammatory cytokines. We generated a double-mutant PRRSV-2 to harbor both mutations and examined for their IFN and NF-κB phenotypes in porcine alveolar macrophages after co-infection with *Streptococcus suis* (*S*. *suis*). Subsequently, the clinical and immunological profiles of the mutant virus were examined in the natural host animals upon co-infection with *S*. *suis*. Our results showed indeed that the double mutant PRRSV-2 exhibited elevated IFN responses and reduced inflammatory cytokines and chemokines in pigs. Furthermore, the double-mutant virus was clinically attenuated in pigs, as shown by the reduced clinical severity, lower viral titers, and shorter duration of viremia, compared to those in pigs co-infected with wild-type PRRSV-2 and *S*. *suis*. Our study highlights that the reprogramming of viral immune evasion is possible, which can be developed as a new strategy for a design of next-generation vaccines.

## Results

### Mutations in the SAP motif of PRRSV-2 nsp1β and PIAS1-binding motif of N confer IFN suppression-negative and NF-κB activation-negative

**Fig 1:**
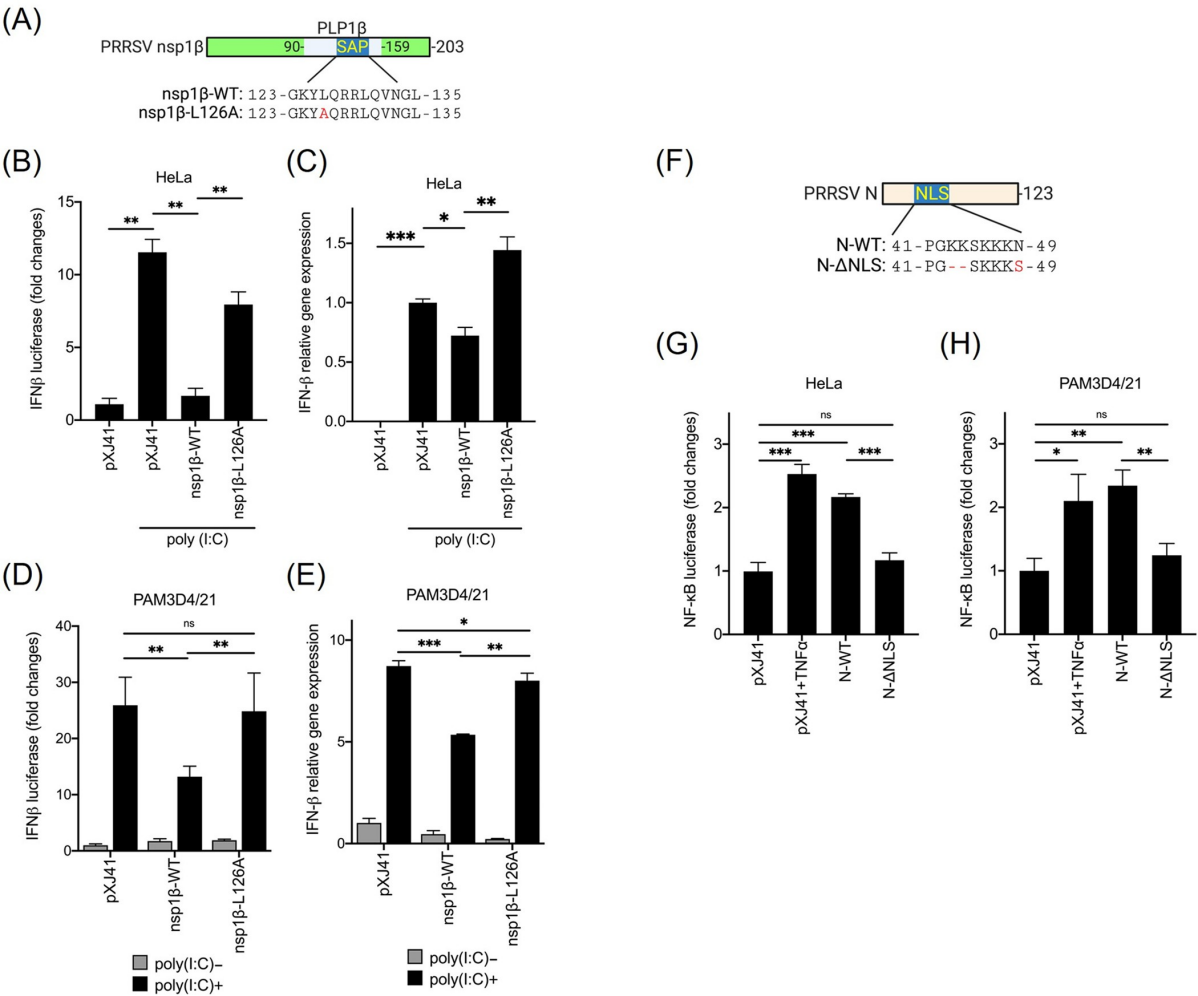
Mutations in the SAP motif of PRRSV nsp1β (A) and the PIAS1-binding motif of N (F) result in the loss of IFN suppression and NF-κB activation, respectively. (A), The functional domains of PRRSV-2 nsp1β. Grey box indicates the papain-like proteinase (PLP) domain, and blue box indicates the SAP motif. Leucine at 126 in the SAP motif of nsp1β was substituted to alanine to generate nsp1β-L126A. HeLa (B) or PAM 3D4/21 (D) cells were cotransfected with pRL-TK and IFN-β-luc along with a respective viral gene and stimulated with poly(I:C) for 12 h. The firefly and Renilla luciferase activities were determined. For IFN-β gene expressions, HeLa (C) or PAM 3D4/2 (E) cells were grown in 6-well plates and transfected with a target gene followed by RT-qPCR at 24 h post-transfection. Swine β-actin mRNA served as a loading control. (F), The nuclear localization signal (NLS) is located at positions 41–47 of the N protein. N-ΔNLS was generated by deleting two lysine residues at 43 and 44 and substituting asparagine at 49 with serine residue in NLS of N to result in PG—SKKKS. (G) HeLa or (H) PAM 3D4/21 cells were co-transfected with the pRL-TK and NF-κB-luc plasmids along with a respective gene and stimulated with TNF-α for 6 h. The firefly and Renilla luciferase activities were determined. Error bars, mean ± standard deviation (s.d.). *, P<0.05; **, P<0.01; ***, P<0.001.

The PRRSV-2 nsp1β protein contains a highly conserved sequence at positions 124–135 among all strains, and this sequence of 124-KxLQxxLxxxGL-135 (where x for any amino acid) resembles the eukaryotic SAP consensus motif (Fig 1A) [46]. Previously, we have shown in monkey kidney epithelial cells that the SAP motif of nsp1β is associated with type I IFN suppression [32,33]. To confirm and validate the IFN regulation by the SAP motif of nsp1β, porcine pulmonary alveolar macrophage (PAM)-originated 3D4/21 cells or Cl3 cells, which are natural target cells of PRRSV, were used to express the L126A nsp1β mutant. IFN productions were then examined using IFN-β-luciferase reporter assay. In this assay, the reporter expression reflects the transcriptional activity of the IFN-β promoter and represents the levels of IFN production. Previously, various IFN assays were compared to each other, including RT-qPCR for IFN mRNAs, vesicular stomatitis virus (VSV)-based bioassay for IFN proteins, and luciferase reporter assay for IFN expressions. VSV is extremely sensitive to type I IFNs, and in the presence of IFNs, VSV replication is inhibited, which can quantitatively be measured [47]. All three assays were comparable and reliable to replace the ELISA-based immunoassay, and thus RT-qPCR and luciferase reporter assays were employed. HeLa cells were co-transfected with wild-type nsp1β (nsp1β-WT) or SAP-motif mutant nsp1β (nsp1β-L126A) along with pIFN-β-luc reporter, followed by quantification of luciferase expressions. Poly(I:C) stimulation increased the luciferase activity by 11.54 folds in empty vector (pXJ41)-transfected cells, whereas the reporter was not increased in nsp1β-WT expressing cells even after stimulation. In contrast, nsp1β-L126A mutant elicited the reporter activity 4.9-folds higher than nsp1β-WT upon poly(I:C) stimulation (Fig 1B), indicating the loss of nsp1β-mediated IFN suppression. To further assess the IFN regulation by nsp1β, total RNA was isolated from wild-type nsp1β- or nsp1β-L126A-expressing cells, and RT-qPCR was conducted. As shown in Fig 1C, nsp1β-WT induced a significantly lower level of IFN-β gene expression than empty vector, while nsp1β-L126A did not suppress IFN-β gene expression after poly(I:C) stimulation (Fig 1C). Similar results were obtained in PAM 3D4/21 cells that the nsp1β-WT protein downregulated both the luciferase reporter and IFN-β gene expressions, whereas nsp1β-L126A was unable to suppress the IFN-β and reporter expressions (Fig 1D and 1E). These results show that the L126 mutation in nsp1β causes the loss of IFN suppression and reverses the IFN antagonism.

During PRRSV-2 infection, the NF-κB signaling is activated, which has been shown in cells and pigs. For the NF-κB activation, the viral N protein has been identified as the main effector [37]. The N protein binds to PIAS1 which is the repressor for NF-κB, and the PIAS1-binding region in N has been mapped to amino acid positions 37–72 which includes the NLS at 41–47 as PGKKNKK. Thus, the PIAS1-binding motif in N was mutated such that two lysine (K) residues at 43 and 44 were substituted with glycine (G) residues. This mutation conferred the loss of NF-kB activation, and the NF-κB activity induced by this mutant was only slightly higher than or not different from that of the empty vector, indicating that the PIAS1-binding motif of N contributes to the NF-κB activity [37]. To further modify this construct, which first two lysine residues at 43 and 44 were deleted and the fourth lysine at 47 was substituted to serine (S), to make PG—NKS. This construct was designated N-ΔNLS. N-ΔNLS was then expressed in cells and examined for the NF-kB activation using the NF-kB promoter-based reporter plasmid pNF-κB-luciferase (Fig 1G and 1H). Cells were co-transfected with the pNF-κB-luciferase reporter, pRL-TK as an internal control, and individual viral genes, and their relative luciferase activities were obtained after normalizing the firefly luciferase to Renilla luciferase activities. The pXJ41 plasmid was included as an empty vector control without TNF-α treatment, and this value set the baseline (value = 1). HeLa cells were treated with TNF-α for 6 h before lysis, which was then used as a positive control. As shown in Fig 1G, the TNF-α treatment stimulated the reporter activity by 2.53 folds, while N-WT induced the reporter activity by 2.17 folds. In contrast, N-ΔNLS was unable to elicit a higher reporter activity (1.17 folds) (Fig 1G). Similarly, N-WT stimulated the reporter activity by 2.34 folds, while N-ΔNLS did not increase the reporter expression in PAM 3D4/21 cells (Fig 1H). Together, our data validate that the PIAS1-binding-motif mutation in N confers the loss of NF-κB activation and suggest that the N gene can be modified to generate a new virus whose NF-κB activation function is removed.

### Generation of SAP motif- and NLS motif-double mutant virus

**Fig 2:**
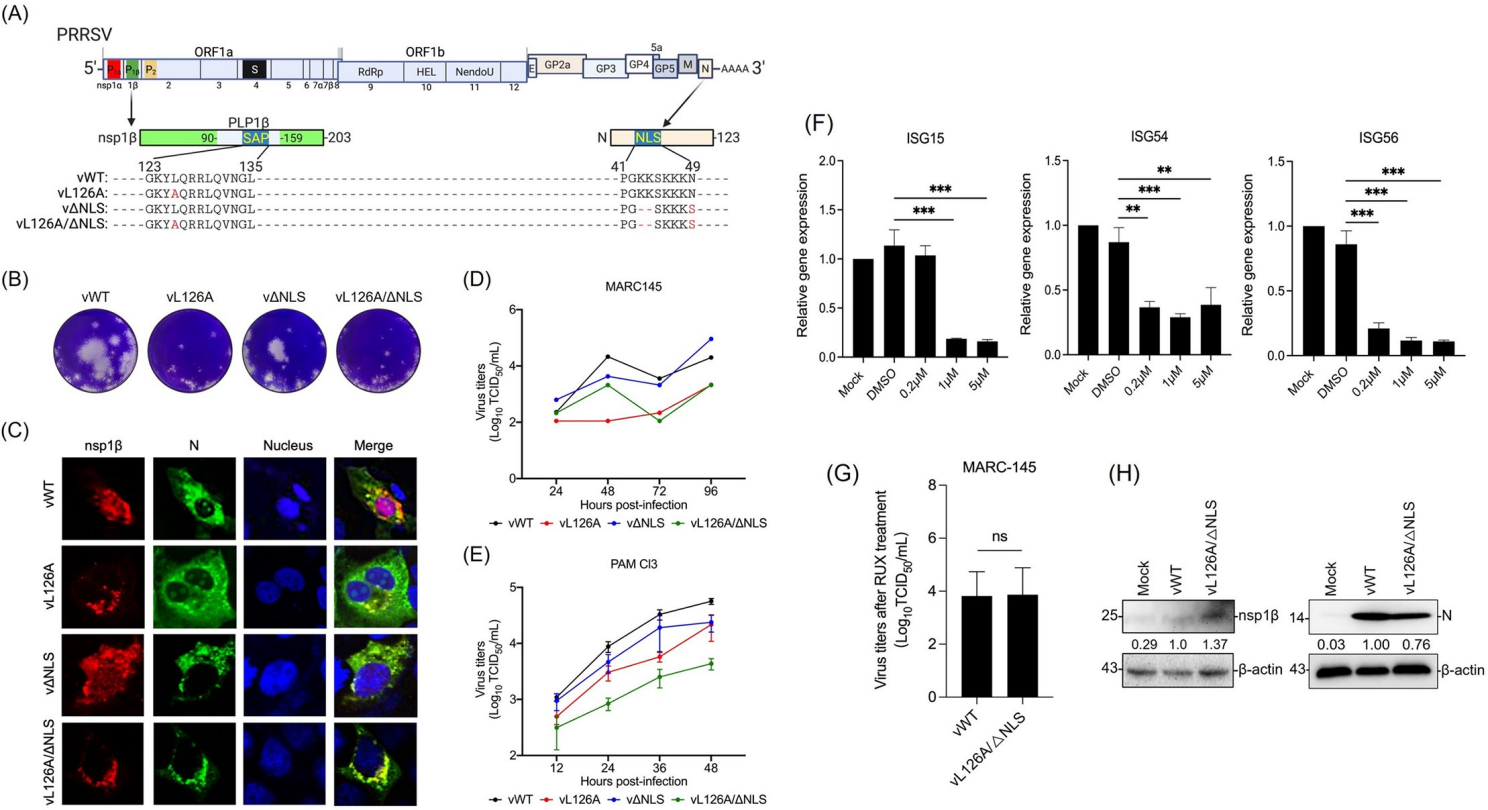
Generation and growth kinetics of PRRSV-2 mutants in MARC-145 cells. (A), Genome organization and constructions of SAP motif nsp1β mutant virus (vL126A), PIAS1-binding motif N mutant virus (vΔNLS), and double-mutant virus (vL126A/ΔNLS) using the P129 infectious clone. Alphabets in red color indicate mutated amino acids. Hyphens in red color indicate amino acid deletions. (B), Plaque morphologies of mutant PRRSVs. (C), Subcellular localization of PRRSV-2 nsp1β (red) and PRRSV-2 N (green) proteins. MARC-145 cells were infected with the respective PRRSV-2 at 1 MOI for 48 h. Cells were stained with α-PRRSV-nsp1β rabbit serum (red) and α-PRRSV-N Mab (green). The nuclei were stained with DAPI (blue). Images were taken by confocal microscopy (Nikon A1R). (D, E), Multistep growth curves for mutant viruses. Cells were infected at 1 MOI in MARC-145 (D) or PAM Cl3 cells, and virus titers were determined as a 50% tissue culture infectious dose (TCID_50_) in MARC-145 cells at indicated times. (F), JAK-inhibitor assay. MARC-145 cells were seeded in 6-well plates and incubated with 1,000 units of human IFN-β for 2 h for stimulation of the JAK-STAT pathway. Then, cells were treated with Ruxolitinib (STEMCELL Technologies, Cambridge, MA) at indicated concentrations. 24 h later, total cellular RNA was extracted, and RT-qPCR was conducted for ISG15, ISG54, and ISG56. The relative fold changes of ISG transcripts were normalized to that of β-actin and statistically analyzed. ***, p<0.001. (G), MARC-145 cells were treated at 1 μM concentration for 24 h and infected with vWT or vL126A/ΔNLS for 48 h. Culture supernatants were collected and titrated by TCID50 in a 96-well plate format by the Reed-Muench method. The experiment was conducted twice in duplicate each. (H), PAM-CL3 cells were treated with Ruxolitinib for 24 h at a concentration of 1 μM and infected with vWT and vL126A/△NLS. At 48 h post-infection, cell lysates were prepared, and Western blot was performed using rabbit anti-N pAb (Novus Biologicals, Centennial, CO) and β-actin mouse mAb.

**Fig 3:**
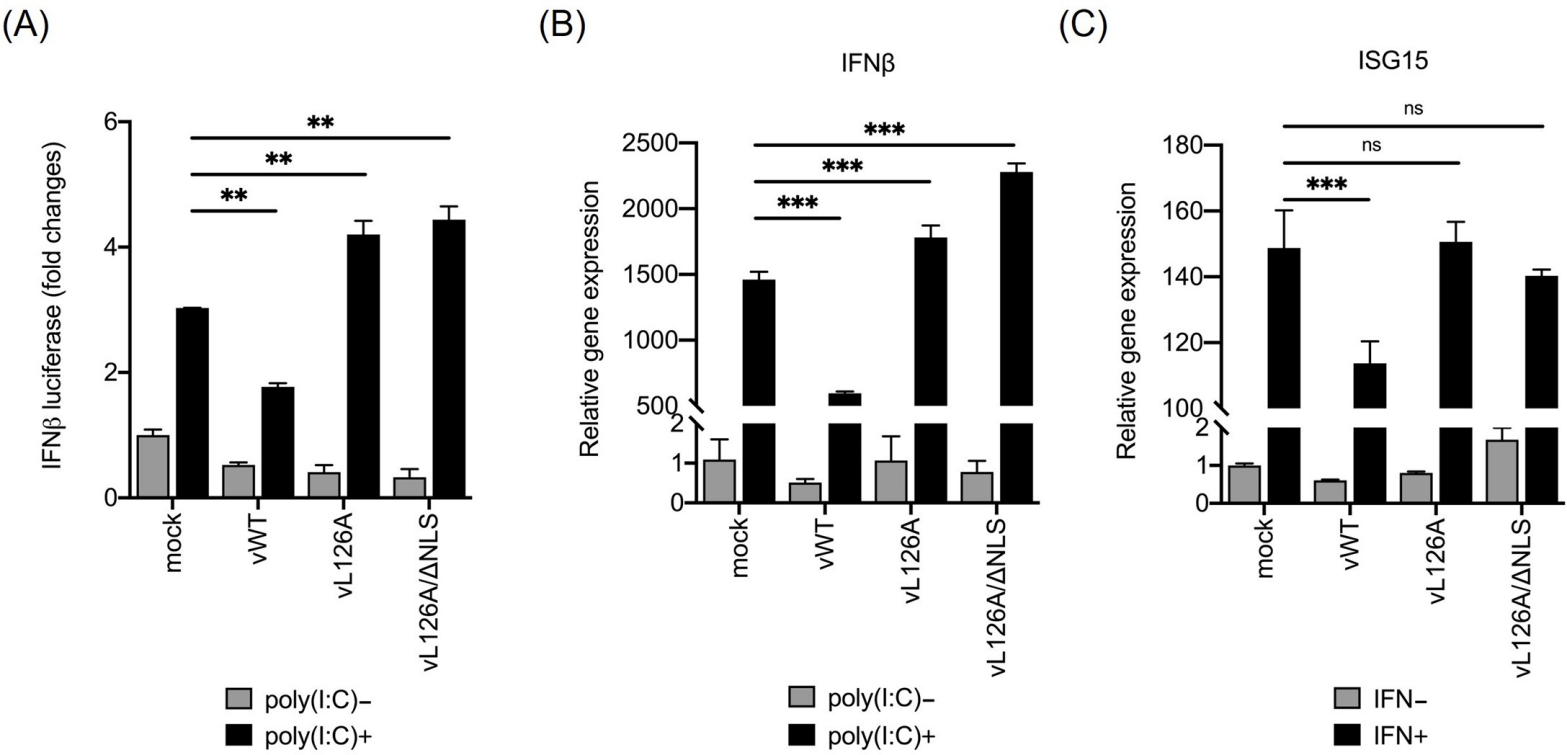
Regulation of type I IFN production by nsp1β SAP mutant PRRSV-2. (A), PAM Cl3 cells were transfected with the pIFN-β-luc reporter, infected with a respective mutant virus 24 h post-transfection (hpi), and subjected to luciferase assays to determine their IFN-β induction. (B), PAM Cl3 cells were infected with a mutant virus and stimulated with poly(I:C) at 24 hpi for 6 h, and then IFN-β mRNA was examined by RT-qPCR. (C), PAM Cl3 cells were infected with a mutant virus, and ISG15 mRNA was examined by RT-qPCR after IFN stimulation. Error bars, mean ± standard deviation (s.d.). ns: no significant difference, **, P<0.01; ***, P<0.001.

Since both the type I IFN suppression and NF-κB activation functions can be modified by mutating the nsp1β and N genes, respectively, as shown in the ectopic gene expression system (Fig 1), it was hypothesized that a mutant PRRSV-2, whose genes for the SAP motif and the NLS motif were individually altered, might lose immunomodulatory functions with its anticipated phenotypes of type I IFN suppression-negative and NF-κB activation-negative. To test this hypothesis, mutant viruses were generated using the infectious cDNA clone of PRRSV-2 strain P129. The vL126A mutant virus was generated by substituting the leucine at 126 to alanine in the SAP motif of the nsp1β. For vΔNLS virus, the mutation described above was introduced to the N gene of the infectious clone. Then, a double-mutant virus was generated and designated vL126A/ΔNLS (Fig 2A). The PRRSV-2 mutants were rescued and amplified in MARC-145 cells by four subsequent passages, and their genetic stability for mutated sequences were confirmed by sequencing. The viral infectivity was determined by cytopathic effects (CPE) (Fig 2B) and immunofluorescence assays (IFA) for the nsp1β and N protein expressions (Fig 2C). The plaques of the vL126A and vL126A/ΔNLS mutants were significantly smaller than those of wild-type virus vWT and vΔNLS at five days, indicating the slower growth of vL126A and vL126A/ΔNLS than vWT and vΔNLS. The multi-step growth kinetics further revealed that vL126A and vL126A/ΔNLS exhibited decreased growth rates with peak titers approximately 1 log lower than that of vWT in MARC-145 cells (Fig 2D). The multi-step growth kinetics were determined for mutant viruses in PAM Cl3 cells. vL126A/ΔNLS displayed reduced growth rates with 1 log lower than that of vWT at 24 hpi through 48 hpi (Fig 2E). The IFN suppression by nsp1β is non-essential for virus replication, but the removal of this function negatively affects the growth of the virus, presumably due to increased production of type I IFN antiviral cytokines (Also see Fig 3).

To address this hypothesis, a JAK-STAT inhibitor experiment was conducted. Ruxolitinib is a potent JAK inhibitor, and we first examined the expression of three representative ISGs at various concentrations of the inhibitor. At 1 μM concentration of Ruxolitinib, the expressions of ISG15, ISG54 and ISG56 were all inhibited compared to the control (Fig 2F). We then examined the replication of the double-mutant virus vL126A/ΔNLS in the presence of Ruxolitinib. By 48 h post-infection, the titer of vL126A/ΔNLS reached a similar level to the wild-type PRRSV-2 (Fig 2G). The titers of both vWT and vL126A/ΔNLS were 1 x 10^4 /ml, and these titers were comparable to each other. We also determined the relative productions of two viral proteins nsp1β and N in virus-infected cells by Western blot (Fig 2H). Taken together, our data indicate that the reduced titers of vL126A/ΔNLS observed in Fig 2D and 2E were indeed due to the alleviation of innate immune suppression and induction of inflammation, not due to the intrinsic property of the mutant virus derived from the viral manipulation. These findings also suggest that IFN suppression is a significant benefit for PRRSV replication.

### Type I IFN suppression of the nsp1β SAP mutant PRRSV *in vitro*

To examine the IFN suppression by mutant PRRSVs, PAM Cl3 cells were infected with vWT, vL126A, or vL126A/ΔNLS, followed by stimulation with poly(I:C) or porcine-specific recombinant IFN-β. IFN activities were examined using IFN-β-luciferase reporter assays in virus-infected cells. PAM Cl3 cells were first transfected with the pIFN-β-luc reporter and after 24 h later, infected with a mutant virus followed by luciferase determination. In mock-infected cells, poly(I:C) stimulation resulted in a 3-fold increase of reporter, whereas vWT-infected cells showed a lower level IFN-β expression even after stimulation. In contrast, vL126A and vL126A/ΔNLS were able to elicit 4.2-fold and 4.4-fold higher levels of IFN-β expression upon poly(I:C) stimulation, respectively (Fig 3A). To further assess the IFN production and JAK-STAT signaling response in mutant virus-infected cells, total RNA was extracted at 24 hpi and analyzed by RT-qPCR for the expression of IFN-β and ISG15 transcripts. In mock-infected cells, poly(I:C) stimulation resulted in an increase of IFN-β mRNA, whereas in vWT-infected cells, IFN-β transcription was significantly lower than that of mock-infected group, indicating the suppression of IFN-β gene expression by vWT as expected. In contrast, both vL126A and vL126A/ΔNLS mutants elicited higher levels of IFN-β expression than vWT, and the expression levels were even higher when compared with mock-infection with stimulation (Fig 3B). The modulation of the JAK-STAT signaling pathway was also assessed by examining ISG15 transcripts in IFN-β-stimulated cells. Incubation of cells with IFN-β enhanced the ISG15 transcription more than 145 folds in mock-infected cells. While vWT triggered the suppression of ISG15 transcription, both vL126A and vL126A/ΔNLS induced the ISG15 transcription to similar levels in mock-infected cells after the IFN-β stimulation (Fig 3C). These results demonstrate that the nsp1β SAP mutant PRRSV does not further suppress the IFN production and JAK-STAT signaling pathways. These data indicate that the L126A mutation in nsp1β results in the loss of type I IFN antagonism, and their immunological phenotype is IFN suppression-negative.

### NF-κB and cytokine responses to the N mutant PRRSV-2 in PAMs

**Fig 4:**
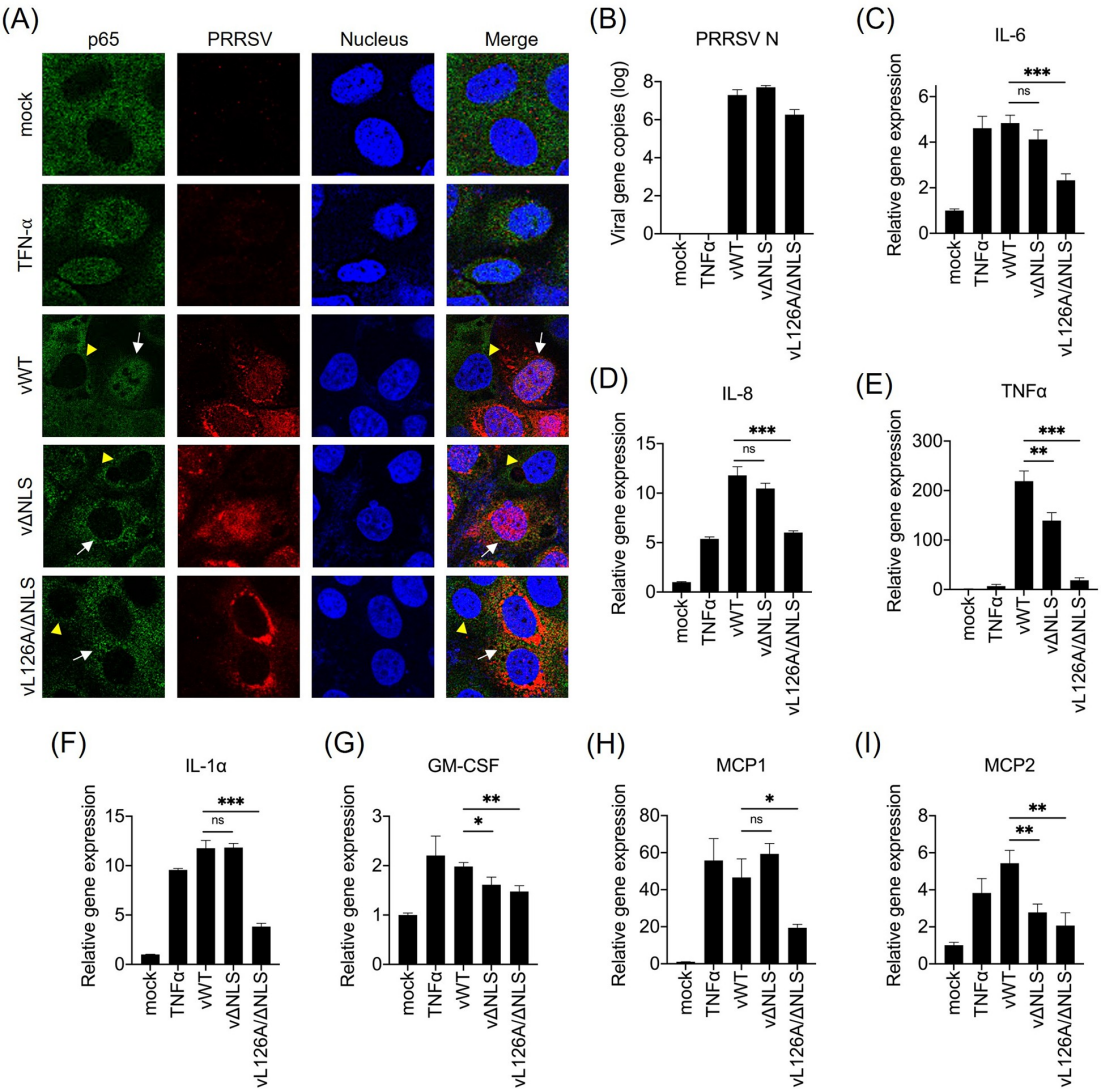
Attenuated expression of immunomodulatory cytokines in PAMs by PIAS1-binding motif mutant PRRSV-2. (A), PAM Cl3 cells were infected with the respective PRRSV at 1 MOI for 24 h. For positive control group, cells were treated with TNF-α (20 ng/ml) for 6 h. Cells were stained with α-PRRSV-nsp1β rabbit serum (red) and α-p65 PAb (rabbit) (green). The nuclei were stained with DAPI (blue). Images were taken by confocal microscopy (Nikon A1R). White arrows indicate the virus-infected cells, and yellow arrows head indicate uninfected cells. (B through I), PAM Cl3 cells were infected with PRRSV-2 at 1 MOI for 24 h or treated with TNF-α (20 ng/ml) for 6 h, and total RNAs were isolated. RT-qPCR was performed to detect PRRSV-N mRNA (B) and transcripts for IL-6 (C), IL-8 (D), TNF-α (E), IL-1α (F), GM-CSF (G), MCP1 (H), and MCP2 (I). The relative amounts of transcripts were calculated using the 2^−ΔΔ^CT method by normalizing the values to that of β-actin. Error bars, mean ± standard deviation (s.d.). ns: no significant difference. *, P<0.05; **, P<0.01; ***, P<0.001.

The PIAS1-binding motif in the N gene of PRRSV-2 has previously been demonstrated to play a crucial role in activating NF-κB and promoting proinflammatory cytokine productions [37]. The activation of NF-κB is dependent on the translocation of p65 (RelA) to the nucleus, and therefore, it was deemed necessary to confirm the NF-κB activation by examining the subcellular distribution of p65 in PIAS1-binding motif-knockout mutant virus vL126A/ΔNLS-infected cells (Fig 4). The results confirmed that p65 was predominantly localized in the nucleus in vWT-infected cells, similar to that observed with TNF-α stimulation. Conversely, in vΔNLS and vL126A/ΔNLS-infected cells, p65 was mostly distributed in the cytoplasm (Fig 4A), indicating the absence of NF-κB activation. To validate this function in the context of virus infection in natural target cells, NF-κB-mediated proinflammatory cytokine gene expressions were quantified in PAM Cl3 cells by RT-qPCR at 24 hpi (Fig 4C). PRRSV N mRNA was first examined in virus-infected cells, and the results showed 1 log lower expression of the viral gene for vL126A/ΔNLS than for vWT and vΔNLS (Fig 4B). When NF-κB-directed proinflammatory cytokine expressions were examined (Fig 4C), vWT significantly upregulated the IL-6, IL-8, and TNF-α transcriptions in PAM Cl3 cells. In contrast, the transcription levels of IL-6 (P < 0.001, ***), IL-8 (P < 0.001, ***), and TNF-α (P < 0.001, ***) were significantly lower in PAM Cl3 cells infected with vL126A/ΔNLS compared to vWT infection (Figs 4C–4E). We also determined the expression of other inflammatory cytokines in virus-infected cells (Fig 4F and 4G). The results showed that vL126A/ΔNLS infection lowered the transcriptions for IL-1α (P < 0.001, ***) and GM-CSF (P < 0.01, **) compared to vWT infection. For the chemokine response to virus infection, MCP1 and MCP2 expressions were significantly reduced (P < 0.05, *, and P< 0.01, **, respectively) in vL126A/ΔNLS-infected cells, compared to those of vWT infection. These findings indicate that vL126A/ΔNLS downregulates NF-κB activation and accordingly reduces the expression of proinflammatory cytokines and chemokines during infection.

### Co-infection of macrophages with PRRSV-2 mutants and *Streptococcus suis* and proinflammatory cytokine responses *in vitro*

**Fig 5:**
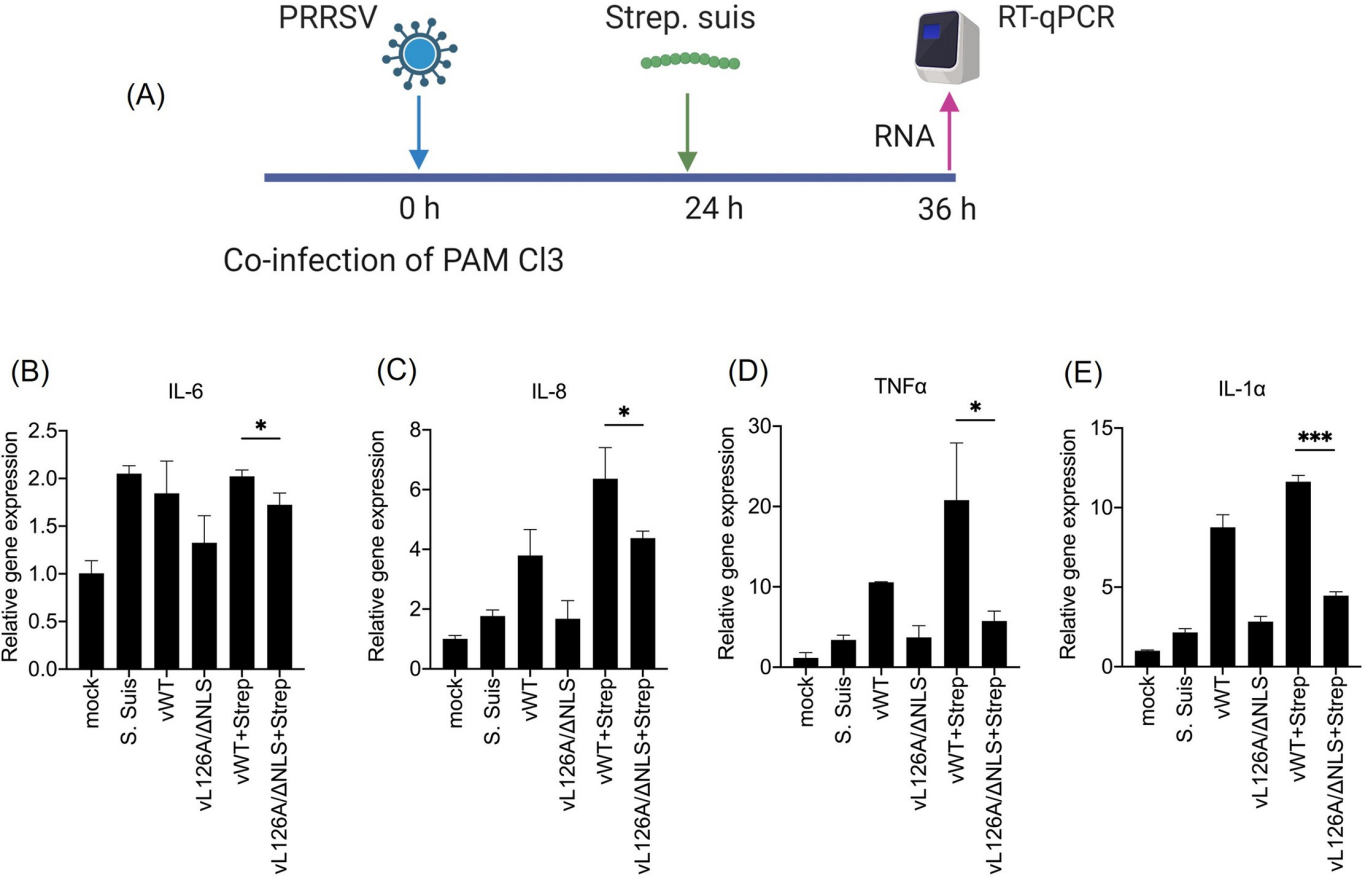
Attenuated production of proinflammatory cytokines by PIAS1-binding-motif N mutant PRRSV-2 in PAMs coinfected with *Streptococcus suis* (*S*. *suis*). (A), Co-infection model of PRRSV-2 and *S*. *suis* in PAM Cl3 cells. PAM Cl3 cells were infected with 1 MOI of PRRSV-2 and, at 24 hpi, inoculated with 10 MOI of *S*. *suis*, followed by quantification of specific transcripts by RT-qPCR at 36 hpi. (B through E), The transcripts for IL-6 (B), IL-8 (C), TNF-α (D), and IL-1α (E) were determined in coinfected cells. The relative levels were calculated using the 2^−ΔΔ^CT method by normalizing the values to that of β-actin. Error bars, mean ± standard deviation (s.d.). *, P<0.05; ***, P<0.001. Panel (A) was created with BioRender.com.

Co-infection of pigs with multiple pathogens is common in swine farms causing PRDC, and PRRSV is the crucial agent. Other pathogens contributing to PRDC include porcine circovirus, swine influenza virus, *Mycoplasma hyopneumonia*, *Actinobacillus pleuropneumonia*, *Pasteurella multocida*, *Streptococcus suis*, and *Hemophillus parasuis* [48,49]. Previous studies showed that co-infection of PRRSV and *S*. *suis* induced severe clinical symptoms accompanied by NF-κB activation and enhanced the production of proinflammatory cytokines in cells and pigs [38,50,51]. Since the vL126A/ΔNLS mutant induced decreased levels of proinflammatory cytokines in PAM Cl3 cells compared to those of vWT (Fig 4), this mutant virus was expected to relieve the hyperexpression of proinflammatory cytokines during co-infection of PRRSV-2 and a secondary pathogen. To examine this hypothesis, PAM Cl3 cells were infected with vWT or vL126A/ΔNLS and, at 24 hpi, inoculated with *S*. *suis*, followed by quantification of specific transcripts at 36 hpi (Fig 5A). As shown in Figs 5B–5E, significant increases of IL-6, IL-8, TNFα, and IL-1α were observed in the vWT and *S*. *suis* co-infection group, compared to the vWT single-infection group, indicating that co-infection of PRRSV-2 and *S*. *suis* enhanced the proinflammatory cytokine expressions. In contrast, the co-infection of vL126A/ΔNLS with *S*. *suis* induced significantly lower-level expressions for IL-6, IL-8, TNFα, and IL-1α in comparison with the vWT and *S*. *suis* co-infection. These results demonstrate that vWT had the ability of NF-κB activation and, during co-infection with *S*. *suis*, produced synergistic expression of cytokines. On the contrary, the vL126A/ΔNLS mutant induced only a marginal elevation of cytokine productions during co-infection with a secondary bacterium.

### Co-infection of natural host animals with PRRSV-2 mutants and *Streptococcus suis* and their clinical, pathological, and virological properties

**Fig 6:**
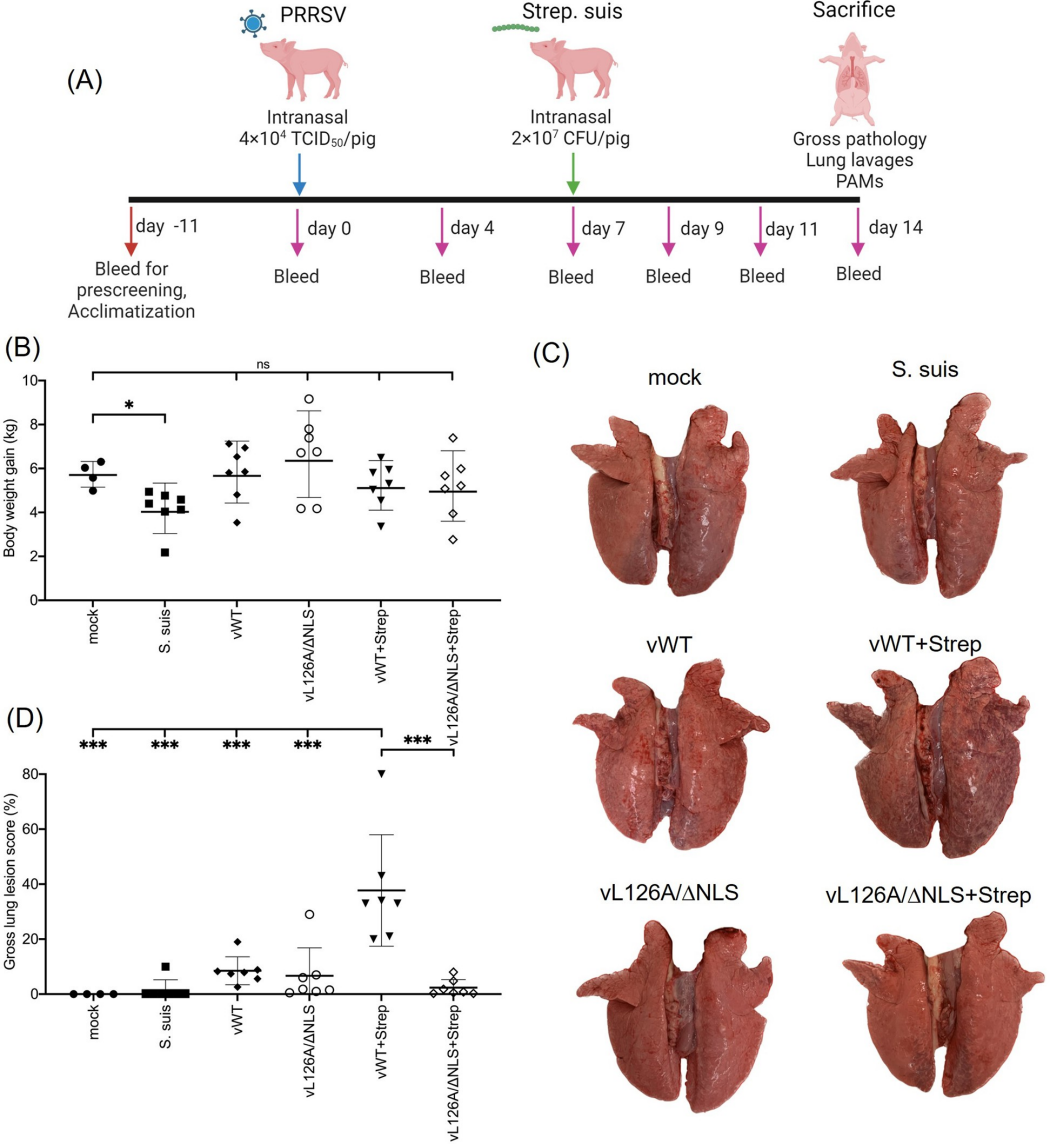
Clinical features of pigs coinfected with PRRSV-2 mutants and S. suis. (A), Experimental design of the co-infection of PRRSV-2 and *S*. *suis* in pigs. (B), Body weights of pigs were measured at day 0 and day 14, and weight gains of individual pigs are shown. (C), representative gross lung pathology of each infection trial. (D), Gross lung lesion scores were calculated by determining the mean percentage value of each lobe that showed visible signs of pneumonia. Gross lung lesion scores provide estimates of the overall proportion of lung tissues affected by gross visible pneumonia. Each dot represents an individual pig. Error bars, mean ± standard deviation (s.d.). ns: no significant difference. *, P<0.05; ***, P<0.001. Panel (A) was created with BioRender.com.

To investigate the immunological and pathogenetic characteristics in the natural host animals after infection with the vL126A/ΔNLS mutant virus, a swine experiment was conducted. A total of 39, 5-week-old piglets were randomly allotted to 6 groups. The mock-control group consisted of 4 animals, while the remaining groups had 7 animals per group. The animals were acclimatized for 1 week and tested negative for PRRSV, porcine circovirus, and swine influenza virus. On day 0, all animals were inoculated intranasally with 4×10^4^ TCID_50_/2 ml of PRRSV per pig, and on day 7, pigs were inoculated intranasally with 2×10^7^ CFU of *S*. *suis* per pig. On day 14, all piglets were euthanized, lung pathology was examined, and tissues were collected for further analysis. Blood samples were periodically collected as indicated (Fig 6A). Body weights were measured on day 0 and day 14, and weight gain changes were recorded.

The pigs that were inoculated with only *S*. *suis* showed lower body weight gains than animals in the mock-infection group. However, no significant differences were found in body weight gains between other infection groups and the mock-control group (Fig 6B). Three of the pigs infected with vWT and *S*. *suis* (vWT+Strep) exhibited swollen rear hock joints 5 days after *S*. *suis* challenge. *S*. *suis* was isolated from swabs taken at necropsy from two of these joints. In addition, this group exhibited the highest extent of gross lung pathology (37.7±5.8%) (Fig 6C), which was characterized by a multifocal to diffuse, lobular pattern of reddening that corresponded with a rubbery feel on palpation, consistent with interstitial pneumonia (Fig 6D, second row-right). Conversely, pigs coinfected with vL126A/ΔNLS and *S*. *suis* (vL126A/ΔNLS+Strep) showed no clinical signs of a systemic *S*. *suis* infection (Fig 6D, third row-right), and exhibited a much lower extent/severity of pneumonic changes (2.3±5.8). The extent of gross lung pathology exhibited by the lungs of pigs in all other treatment groups was not statistically different from the strict control group (Fig 6C). Histologically, specimens taken from pneumonic lungs demonstrated varying severities of histiocytic interstitial pneumonia, characterized by expansion of the alveolar septa by mononuclear inflammatory cells (histiocytes/macrophages), alveolar exudate consisting of necrotic debris and macrophages, and atelectasis with resultant tissue consolidation.

**Fig 7:**
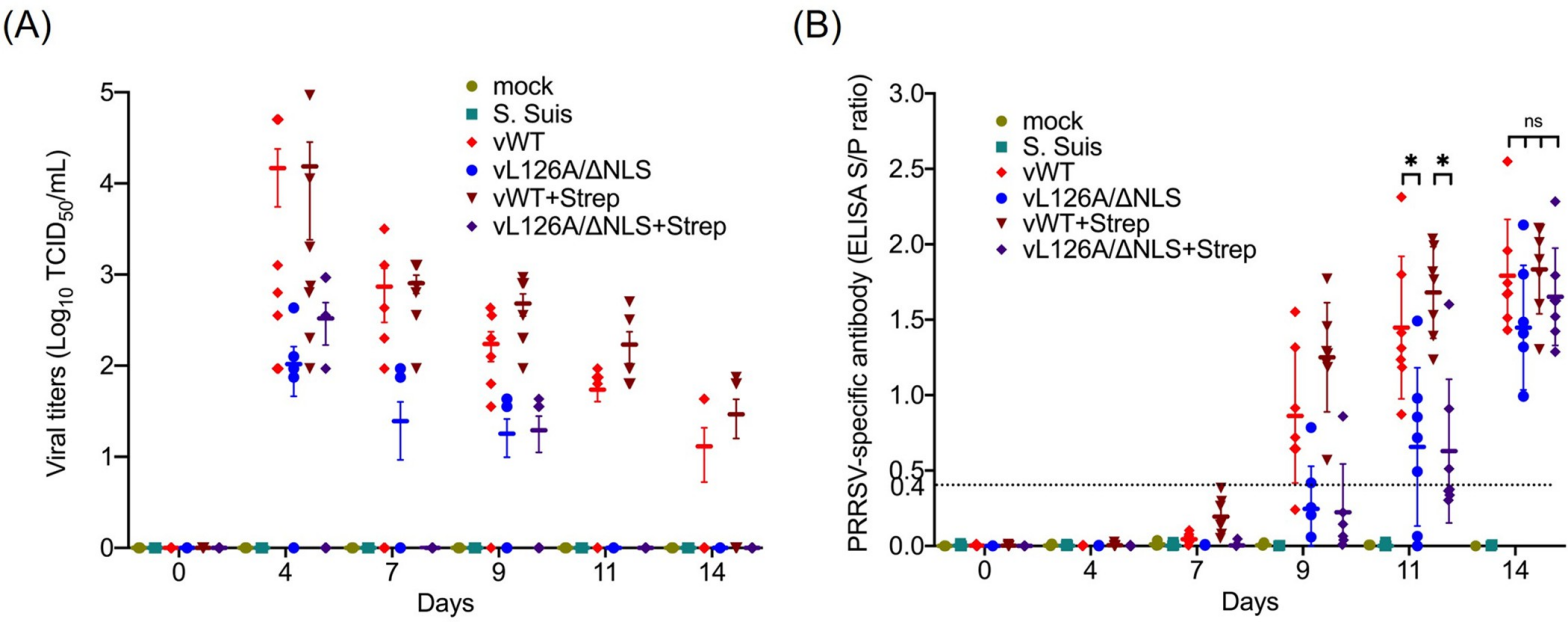
Virological characteristics of pigs coinfected with PRRSV-2 mutants and S. suis. (A), Viral titers were determined in the sera of pigs at the indicated times. 50% tissue culture infectious dose (TCID_50_) was determined in triplicate using MARC-145 cells, and the viral titers was calculated as TCID_50_/mL of serum. (B), PRRSV-specific antibody was determined using the IDEXX HerdCheck PRRSV ELISA kit. S/P ratio = 0.4 was set as the cutoff value (dotted line) for positive/negative. S/P ratios greater than 0.4 were considered positive. An individual pig is represented by each dot, and different shapes of symbols in different colors represent different groups. Error bars, mean ± standard deviation (s.d.). ns: no significant difference. *, P<0.05.

All pigs in the vWT and vWT+Strep groups showed viremia by 4 dpi, with an average viral titer of 4.17 and 4.19 log TCID_50_/mL, respectively (Fig 7A). In the vL126A/ΔNLS group, 4 out of 7 pigs became viremic at 4 dpi with a mean titer of 2.26 log TCID_50_/ml. Similarly, in the vL126A/ΔNLS+S. suis group, 4 out of 7 pigs became viremic at 4 dpi, with a mean virus titer of 2.76 log TCID_50_/ml. The viral titers of all groups decreased gradually from 7 dpi. With a viral titer of 2.87 log TCID_50_/ml for the vWT group and 2.90 log TCID_50_/ml for the vWT+Strep group on 7 dpi, only 2 out of 7 pigs remained viremic (1.92 log TCID_50_/ml) for the vL126A/ΔNLS group. All pigs in the vL126A/ΔNLS+Strep group became negative for viremia. On day 9, which was 2 days after *S*. *suis* infection, the vWT/ΔNLS+Strep group showed higher titers of viremia (2.68 log TCID_50_/mL) than the vWT group of 2.30 log TCID_50_/ml. The viremia became negative by day 14 for the vWT and vWT/ΔNLS+Strep groups with 1 pig in the vWT group and 2 pigs in the vWT/ΔNLS+Strep group continued to be viremic. This observation on the clearance of viremia by 2 weeks post-infection was similar to the previous report [52], which led us to ignore the immunohistochemistry and H&E staining for viral antigens in the lungs. Overall, the duration of viremia for the vWT and vWT/ΔNLS+Strep groups was longer with higher titers than for the vL126A/ΔNLS and vL126A/ΔNLS+Strep groups (Fig 7A).

The PRRSV-specific antibody response was also assessed by ELISA (Fig 7B). Throughout the study, both the mock and *S*. *suis* groups remained seronegative, indicating no cross-infection between groups. For the vWT and vWT+Strep groups, all pigs seroconverted by 9 dpi and remained seropositive throughout the study. For the vL126A/ΔNLS group, only 2 pigs out of 7 seroconverted at 9 dpi, and for the vL126A/ΔNLS+Strep group, only 1 out of 7 pigs seroconverted at 9 dpi. By 11 dpi, however, all remaining pigs (5 pigs in the vL126A/ΔNLS group and 4 pigs in the vL126A/ΔNLS+Strep group) became seropositive. Significant differences (P < 0.05) were observed between the average titers (S/P ratio) of antibodies for pigs in the vWT and vL126A/ΔNLS infection groups. All pigs in the PRRSV-infected groups seroconverted by 14 dpi. No statistical differences were identified in antibody titers between all four PRRSV-infection groups (Fig 7B). Pigs infected with vL126A/ΔNLS exhibited lower lung lesions and lower viral titers with a shorter duration of viremia compared to those infected with vWT. No significant differences, however, were identified for PRRSV-specific antibody titers between two groups of pigs.

### Inflammatory cytokines and chemokine responses in co-infected pigs

**Fig 8:**
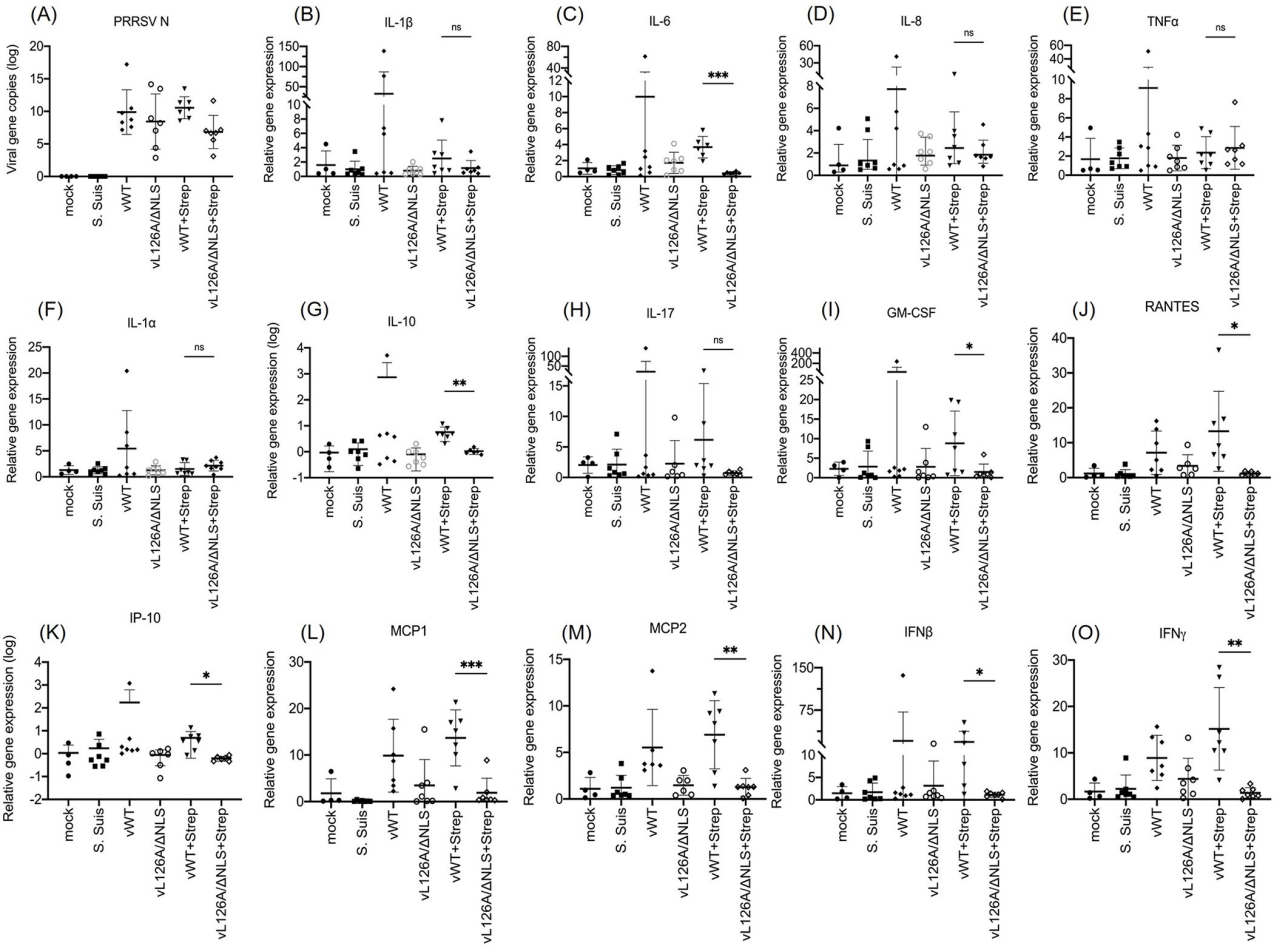
Gene expression profiles for mRNA transcripts of immunoregulatory cytokines and chemokines in cells isolated from bronchoalveolar lavages (BALs) from pigs determined by RT-qPCR. BALs from individual pigs at the time of sacrifice were collected, and cells were isolated which would mostly represent alveolar macrophages. Total RNAs were extracted, and RT-qPCR was conducted to measure specific mRNAs for various genes, including PRRSV-N gene (A) and transcripts for IL-1β (B), IL-6 (C), IL-8 (D), TNF-α (E), IL-1α (F), IL-10 (G), IL-17 (H), GM-CSF (I), RANTES (J), IP-10 (K), MCP1 (L), MCP2 (M), IFN-β (N), and IFN-γ (O). The 2^−ΔΔCT^ method was used to calculate the relative expressions by normalizing the values to that of β-actin. The results were presented as mean ± standard deviation (s.d.) with error bars. *, P<0.05; **, P<0.01; ***, P<0.001.

**Fig 9:**
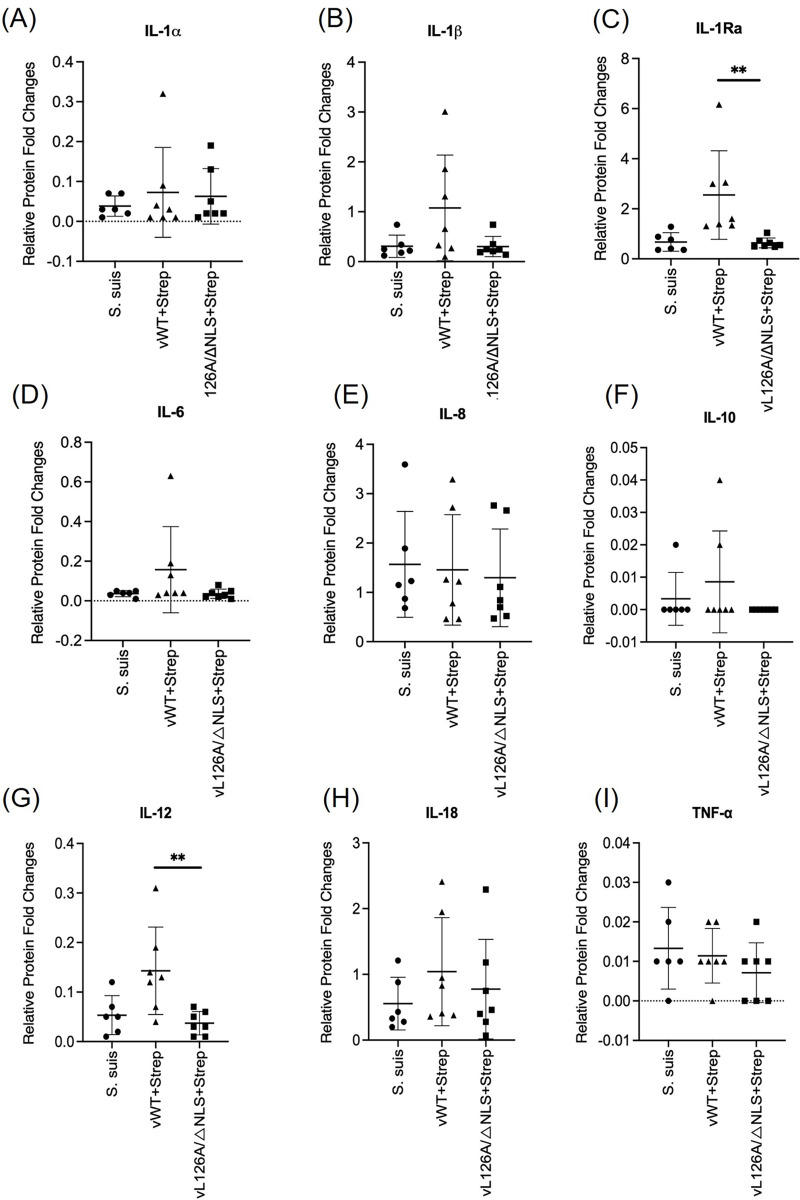
Expression profiles of immunoregulatory cytokines in bronchoalveolar lavages (BALs) collected from pigs coinfected with vWT or vL126A/ΔNLS and Strep. suis. A total of 9 indicated cytokines were determined using the PCYTMG-23K-13PX porcine cytokine immunoassay plate in the MILLIPLEX Multiplex for Luminex Immunoassay system as described in the Materials and Methods. Each sample was analyzed in duplicate. Quality controls and standard controls were established for the porcine cytokine-antibody immobilized magnetic bead panel. Signals were detected using Streptavidin-Phycoerythrin, and the plate was read in the multiplex analyzer (Luminex 200 System, DiaSorin; Stillwater, MN). The data were analyzed using the accompanied software (Luminex SD xMAP Technology LX100/200). **, <0.01.

PRRSV-2 plays a major role in the development of PRDC when coinfected with a secondary pathogen [48], and because NF-κB activation is one of the key features in pigs with PRDC [41–45], the NF-κB activation-negative PRRSV is hypothesized to relieve hyperproduction of cytokines and thus, to attenuate the clinical severity of PRDC in pigs. To examine this hypothesis, we harvested bronchoalveolar lavages (BAL) from individual pigs at the time of sacrifice, and collected cells, most of which would represent alveolar macrophages and some neutrophils. We then determined the relative mRNA transcripts by RT-qPCR and the proteins by immunoassays to represent the expression of inflammatory cytokines and chemokines (Figs 8 and 9). The viral N protein was also determined as the representative viral gene product to ensure the infection (Fig 8A).

The PRRSV-2 N gene expression was detectable in all infection groups except the mock-control group (Fig 8A), confirming that PAMs in all infection groups were infected and responsive. The vL126A/ΔNLS+Strep group exhibited a significantly lower-level expression of IL-6 compared to the vWT+Strep group (Fig 8C) when NF-κB-directed proinflammatory cytokines were examined. However, no significant differences were identified for IL-1β, IL-8, and TNFα (Fig 8B, 8D and 8E) between the vWT+Strep and vL126A/ΔNLS+Strep groups. With regards to immunoregulatory cytokine responses, the vWT+Strep group induced statistically higher-level expressions of IL-10, GM-CSF, IFN-β, and IFN-γ (Fig 8) compared to those of the vL126A/ΔNLS+Strep group, while no statistical difference was found for IL-1α (Fig 8F). The IL-17 transcription also showed an increase in the vWT+Strep group compared to the vL126A/ΔNLS+Strep group although it was statistically insignificant (Fig 8H). Previously, the immunoregulatory chemokines were reported to be upregulated in PRRSV-infected PAMS and were suggested as a major contributor to the pathogenesis of PRRSV-2 infection [53]. In our study, immunoregulatory chemokines were rather decreased for RANTES, IP-10, MCP1, and MCP2 in the vL126A/ΔNLS+Strep group compared to the vWT+Strep group (Fig 8J–8M).

Besides the mRNAs for cytokines, a protein-based assay was conducted to confirm the RT-qPCR data. Due to the numbers of pigs representing different treatment groups and the comparative quantifications of cytokines and chemokines from each pig, Western blots were considered inappropriate for this study. Instead, Luminex porcine-specific cytokine immunoassays were chosen to analyze comparative expressions of inflammatory mediators from lung lavages collected from pigs of coinfection groups (Fig 9). The multiplex immunoassay is analogous to ELISA and can simultaneously detect multiple cytokines. The lower levels of IL-1β, IL-1Ra, IL-6, IL-12, and IL-18 were detected in the lung lavages from pigs of the vL126A/ΔNLS+Strep groups compared to those of the vWT+Strep group (Fig 9B, 9C, 9D, 9E and 9H). In severe and fatal COVID-19 patients, those cytokines were identified as markers for a hypercytokinemia profile according to the meta-analysis [54,55], supporting our data and hypothesis that vL126A/ΔNLS PRRSV reduces the NF-kB-mediated, cytokine storm-like response during coinfection with Strep. suis. The decreased expressions of IL-1Ra and IL-12 in particular were notable and significant. IL-1Ra (interleukin-1 receptor antagonist) is secreted from macrophages, monocytes, and neutrophils, and its expression is stimulated by bacterial or viral components [56]. In mice, the tissue distribution of IL-1Ra is found predominantly in peripheral blood cells, lungs, spleen, and liver. It is a key regulator of the inflammatory responses in a host-specific manner [57]. Although the expression levels of some of inflammatory cytokines were statistically insignificant between the vWT+Strep and vL126A/ΔNLS+Strep groups, their expression levels still showed a tendency of reduction in the vL126A/ΔNLS+Strep. The immunoassay data were largely consistent with the mRNA data, and all together, our data demonstrate that the vL126A/ΔNLS double mutant PRRSV-2 attenuates the proinflammatory cytokine expressions during coinfection with a bacterial pathogen in pigs.

### Genetic stability of the SAP motif in nsp1β and NF-kB regulating domain in N of the mutant viruses *in vivo*

The smaller plaque sizes and lower viral titers of the vL126A/ΔNLS and vL126 mutants implicate the possible presence of strong selection pressure on the IFN-suppression function of PRRSV-2. We postulated that our mutant viruses might have undergone compensatory mutations or sequence changes to recover their evasion function during the infection of pigs. We passaged the mutant viruses in cell culture to examine their genetic stability and sequenced the nsp1β and nucleocapsid protein genes to which two mutations were introduced. Both vL126A/ΔNLS and vL126 were stable in cell culture at least for up to 10 passages. For the N protein gene, reversion was not anticipated and did not occur because a deletion of two amino acids was introduced (Table 1). For nsp1β, a single amino acid substitution was introduced with 2 nucleotide changes to alter the lysine codon (Table 1), and no reversion was observed in cell culture. The stability of the nsp1β mutation in cell culture was thought to be due to the absence of selective pressure in vitro. To examine the evolution of mutant PRRSVs in vivo, the viral RNAs isolated from PAMs of BAL were subjected to RT-PCR and sequencing for the nsp1β and N genes. No single mutation or changes to the wild-type sequence was identified in the N gene for both the vL126A/ΔNLS and vL126A/ΔNLS+Strep groups (Table 1). For the nsp1β gene, however, 4 out of 7 pigs in the vL126A/ΔNLS group and 5 out of 7 pigs in the vL126A/ΔNLS+Strep group undergone mutations from GCU for Ala 126 to GUU for Val 126. In addition, 1 pig in the vL126A/ΔNLS+Strep group acquired a mutation from GCU for Ala 126 to AUU for Ile 126. 3 pigs out of 7 in the vL126A/ΔNLS group and 1 pig in the vL126A/ΔNLS+Strep group did not undergo any sequence changes for the nsp1β gene. The mutation of L126V in the SAP motif of nsp1β was shown to regain nsp1β-mediated type I IFN suppression function in cells, suggesting that vL126A/ΔNLS may have lost the type I IFN suppression by 14 dpi. These results demonstrate the presence of strong selection pressure on the IFN antagonism for PRRSV. Although we had a particular interest in type I IFN response of pigs in the mutant PRRSV-infected groups, PRRSV-2 suppresses IFN response at an early stage of infection, and shortly after, IFNs are bounced back to 20, normal and increased by 3–4 days of infection, which led us to ignore the type I IFN measurements in the present study.

**Table 1:** Reversion of the SAP motif in the nsp1β sequence and the NLS motif in the N sequence.

Group	Pig no.	nsp1β SAP motif		N NLS	
		nucleotide sequence AAGTACCTACAGCGGAGG	reversion	nucleotide sequence CCGGGCAAGAAAAGTAAGAAGAAAAAC	reversion
vL126A/ΔNLS	9	AAGTACGCTCAGCGGAGG	n/a	CCGGGC––––––TCTAAGAAGAAATCC	n/a
	10	AAGTACGCTCAGCGGAGG	n/a	CCGGGC––––––TCTAAGAAGAAATCC	n/a
	24	AAGTACGTTCAGCGGAGG	126A→V	CCGGGC––––––TCTAAGAAGAAATCC	n/a
	25	AAGTACGTTCAGCGGAGG	126A→V	CCGGGC––––––TCTAAGAAGAAATCC	n/a
	31	AAGTACGTTCAGCGGAGG	126A→V	CCGGGC––––––TCTAAGAAGAAATCC	n/a
	34	AAGTACGTTCAGCGGAGG	126A→V	CCGGGC––––––TCTAAGAAGAAATCC	n/a
	36	AAGTACGCTCAGCGGAGG	n/a	CCGGGC––––––TCTAAGAAGAAATCC	n/a
vL126A/ΔNLS+Strep	5	AAGTACGTTCAGCGGAGG	126A→V	CCGGGC––––––TCTAAGAAGAAATCC	n/a
	14	AAGTACGTTCAGCGGAGG	126A→V	CCGGGC––––––TCTAAGAAGAAATCC	n/a
	16	AAGTACGCTCAGCGGAGG	n/a	CCGGGC––––––TCTAAGAAGAAATCC	n/a
	17	AAGTACGTTCAGCGGAGG	126A→V	CCGGGC––––––TCTAAGAAGAAATCC	n/a
	19	AAGTACGTTCAGCGGAGG	126A→V	CCGGGC––––––TCTAAGAAGAAATCC	n/a
	29	AAGTACATTCAGCGGAGG	126A→I	CCGGGC––––––TCTAAGAAGAAATCC	n/a
	40	AAGTACGTTCAGCGGAGG	126A→V	CCGGGC––––––TCTAAGAAGAAATCC	n/a

A-V, alanine to valine mutation; A-I, alanine to isoleucine mutation.

n/a, not applicable.

## Discussion

Porcine respiratory disease complex (PRDC) is a global challenge to the pig industry, and PRRSV is one of the major responsible pathogens [39]. However, development of effective prevention strategies against PRRSV and PRRSV-mediated PRDC has been hindered by the viral antagonism to impair the host immune system and to modulate adequate host response to infections [58,59]. While type I IFNs are potent antiviral cytokines, NF-κB activation and enhanced production of proinflammatory cytokines are considered major effectors for viral pathogenesis both in cells and pigs [44,60,61]. These studies suggest that higher levels of type I IFNs and lower levels of proinflammatory cytokines may render protection against PRRSV-mediated PRDC.

PRRSV-2 nsp1β is a highly potent viral protein that disrupts the type I IFN production and signaling [27–30]. The conserved SAP motif within nsp1β interacts with Nup62, which is crucial for nucleocytoplasmic trafficking of cellular molecules and inhibits type I IFNs by impeding the cytoplasmic translation of host mRNAs [32,62]. In a previous study, a series of SAP mutants that showed the negative phenotype of host mRNA nuclear retention [32]. Among these SAP mutants, replacing the leucine at position 126 to alanine in nsp1β showed the predominant effect on IFN suppression [32]. In the present study, we generated an infectious PRRSV-2 of which phenotype was type I IFN suppression-negative. The plaque size and growth rate of the SAP mutant PRRSV-2 displayed significantly different from those of wild-type PRRSV-2 (Fig 2), indicating that mutation in the SAP motif of nsp1β affects negative effects on the viral replication. The decreased growth of the mutant viruses may be attributed to the increased production of IFNs. A previous study has shown that nsp1β also facilitates the translational frameshifting in the nsp2 region of PRRSV, and the leucine at 126 in the SAP motif resides in the close proximity to key residues that mediate the frameshift expression of nsp2TF and nsp2N [63]. Consequently, it is plausible that the SAP mutant PRRSV-2 may not process nsp2TF and nsp2N normally and further influence the viral replication. In the current study, we demonstrated that the SAP mutant PRRSV-2 lost the suppression function for IFN production and JAK-STAT signaling in PAMs, the natural target cells for PRRSV (Fig 3).

Besides PRRSV, SAP motif mutation and IFN upregulation have been demonstrated for foot-and-mouse disease virus (FMDV). The leader protein L^pro^ of FMDV contains the SAP motif, and the SAP mutant FMDV was attenuated in pigs, as evidenced by the absence of clinical signs and viremia [64]. Additionally, pigs inoculated with the SAP mutant FMDV exhibited a robust immune response, characterized by high levels of neutralizing antibodies. Such an enhanced immune response provided sufficient protection to animals against subsequent challenge with virulent FMDV. Given the pleiotropic effects of type I IFNs on the adaptive immunity, it is plausible that the increased levels of IFNs observed in SAP mutant FMDV-infected pigs can be linked to the elevated antibody response. After removing the IFN suppression function from FMDV, the IFN antagonism-negative virus induced a strong neutralizing antibody response in vaccinated animals, and these animals were completely protected from the high dose virulent challenges. Swine influenza virus (SIV) is another example with a similar outcome. For SIV, NS1 protein is the IFN antagonist, and the mutation in NS1 of SIV caused the loss of viral IFN suppression. The NS1 mutant SIV was clinically attenuated in pigs and stimulated type I IFN production [65]. Furthermore, the pigs immunized with the IFN-suppression-negative SIV conferred the protection against both homologous and heterologous SIV challenges. In the present study, we showed that SAP mutant PRRSV-2 induced lower viral titers and shorter duration of viremia in pigs compared to wild-type PRRSV-2 (Fig 7A). Nevertheless, the pigs infected with SAP mutant PRRSV-2 developed similar levels of PRRSV-specific antibody titers to those of wild-type PRRSV-2 (Fig 7B). Yet, the role of PRRSV-specific antibodies in protection remains unknown. Previously, the passive transfer of serum antibodies conferred partial protection against homologous challenge [66,67]. The development of neutralizing antibodies typically requires multiple exposures to PRRSV over an extended period of time to reach high titers [66–68], limiting us to examine neutralizing antibodies at 14 dpi in the current study. Based on the previous findings, however, it is tempting to speculate that immunization of pigs with the SAP mutant PRRSV may result in an improved protection.

Viruses can exploit the NF-κB signaling pathways to facilitate their own replication [69,70]. The replication of PRRSV in porcine macrophages produces endoplasmic reticulum stress triggering an unfolded protein response resulting in the activation of NF-κB and the production of TNF-α [71]. PRRSV infection in pigs results in an increased expression of proinflammatory cytokines and leads to the recruitment of monocytes and neutrophils to the lungs, thereby contributing to the pathogenesis of PRRSV [72]. PRRSV activates NF-κB in infected pigs, which in turn triggers the production of NF-κB-dependent proinflammatory cytokines [73,74]. Studies have shown that highly pathogenic (HP)-PRRSV enhanced NF-κB activation more than typical PRRSV, emphasizing the crucial role of NF-κB in the viral pathogenesis for PRRSV [36]. The viral N protein is so far the only known PRRSV-2 protein that activates NF-κB [37]. Specifically, the region between residues 37 and 72 of N is known to interact with PIAS1, which releases p65 and frees up NF-κB from the repressor. The mutation of PIAS1-binding region from N abrogates its ability for NF-κB activation [37]. The PIAS1-binding region in N includes NLS motif, and previously, a series of NLS-mutant PRRSVs was generated and examined for their clinical outcome in pigs. These NLS mutant PRRSVs were clinically attenuated in pigs, indicating a correlation between the nuclear localization of N and the clinical severity of PRRSV [75,76]. In the current study, we rescued a PIAS1-binding motif-mutant PRRSV-2, and this mutant virus showed reduced expressions of NF-κB-mediated cytokines, especially for IL-6, IL-8, and TNF-α in PAM Cl3 cells compared to wild-type PRRSV-2 (Fig 4). This observation indicates that the mutation in the PIAS1-binding domain of N suppresses the production of proinflammatory cytokines in the context of PRRSV infection in natural target cells.

Activation of NF-κB and subsequent increase of proinflammatory cytokines are major effectors on pathogenesis during co-infection of PRRSV-2 with secondary microbial pathogens such as *S*. *suis* and *H*. *parasuis* [43,44]. *S*. *suis* is a pathogen commonly observed with PRRSV in clinical cases, and co-infection of PRRSV-2 with *S*. *suis* can lead to exacerbated clinical disease, resulting in increased morbidity and mortality in pigs [61]. The findings from our study support these observations. Specifically, wild-type PRRSV-2 increased activation of NF-κB and enhanced induction of IL-6, IL-8, and TNF-α in coinfected cells compared to single infection with *S*. *suis* (Fig 5). In contrast, the expression levels of IL-6, IL-8, and TNF-α were decreased in cells coinfected with vL126A/ΔNLS and *S*. *suis* compared to wild-type PRRSV-2 and *S*. *suis* co-infection. These results suggest that PIAS1-binding motif-mutant PRRSV lacks the ability to induce NF-κB and proinflammatory cytokines.

Multiple studies have shown enhanced cytokine expressions in cells and pigs when coinfected with PRRSV and various bacterial pathogens. Co-infection of monocytes with PRRSV-2 strain IAF-Klop and *S*. *suis* led to synergistic effects on the expressions of IL-6, CCL5, and TNF-α, and additive effects on productions of CCL4, CCL14, CCL20, IL-15, and PTGS2 (COX-2) [43]. Additionally, co-infection of pigs with NADC30-like PRRSV-2 strain SDlz1601 and *S*. *suis* upregulated IL-1β, IL-6, IL-8, TNF-α, CCL4, IL-10, and INF-β in PAMs [60]. In other studies using *H*. *pararsuis* and PRRSV-1 strain CAPM V-490, IL-1β, IL-8, TNF-α, CD80, and IL-10 expressions were increased in PAMs [77]. IL-1β and TNF-α were upregulated in PAMs of pigs coinfected with *H*. *pararsuis* and HP-PRRSV-2 strain HuN4 [44]. Co-infection of PAMs with HP-PRRSV-2 strain NJGC and *M*. *hyopneumonia* increased IL-1β expression by more than 10 folds [78]. We also identified in the current study that co-infection of pigs with vL126A/ΔNLS and *S*. *suis* decreased expression of immunomodulatory cytokines and chemokines (Fig 8). Notably, our study is the first to use a mutant PRRSV-2 for co-infection in PAMs and pigs and to show the deletion of PIAS1-binding motif from N attenuate proinflammatory cytokine productions in pigs.

In summary, the double-mutant PRRSV-2 lacking both SAP in nsp1β and PIAS1-binding motifs in N was successfully generated. This virus exhibited type I IFN suppression-negative and NF-κB activation-negative and resulted in attenuated production of proinflammatory cytokine in cells. Furthermore, this double-mutant PRRSV-2 reduced clinical severity during co-infection with a secondary bacterial pathogen. Our study paves a way to the development of a new vaccine candidate aiming to reduce the clinical severity of PRDC.

## Materials and methods

### Ethics statement

The animal study protocol was approved by the Institutional Animal Care and Use Committee (IACUC) of the University of Illinois at Urbana-Champaign.

### Cells, viruses, bacteria

HeLa (NIH HIV Reagents Program, Germantown, MD) and MARC-145 cells were maintained in Dulbecco’s modified Eagle’s medium (DMEM; Corning Inc., Corning, NY) supplemented with 10% heat-inactivated fetal bovine serum (FBS; Gibco, Grand Island, NY). 3D4/21 (ATCC CRL-2843) cells that are SV40 large T-transformed porcine alveolar macrophages were cultivated in RPMI 1640 medium (Gibco, Grand Island, NY) supplemented with 10% heat-inactivated FBS in a humidified incubator with 5% CO2 at 37°C. PRRSV-2 (North American type) strain PA8 was propagated in MARC-145 cells and used as a virus stock. PRRSV-2 strain P129 was reconstituted from the P129 infectious clone (51). The reconstituted virus was designated vWT and used as a wild-type virus control for co-infection studies in cells and pigs, which implicates that the current findings may be limited to PRRSV-2. PAM Cl3 is an immortalized porcine alveolar macrophage cell line developed by Y. Lee, Utah State University (Logan, UT) and was cultivated in RPMI 1640 medium (Gibco) supplemented with 10% heat-inactivated FBS, 1X MEM Non-Essential Amino Acids Solution (Gibco), and 250 μg/mL of G418 sulfate (Corning Inc.) in a humidified incubator with 5% CO_2_ at 37°C. *Streptococcus (S*.*) suis* was originally isolated from the porcine brain submitted as clinical specimens to the Veterinary Diagnostic Laboratory (VDL), University of Illinois at Urbana-Champaign (UIUC; Urbana, IL), and was kindly provided by Chien-Che Hung at VDL. *S*. *suis* was grown on Tryptic Soy Agar (TSA, Difco Laboratories) containing 5% FBS for 12 h. Then, the bacteria were washed with PBS using centrifugation, and the final concentration was adjusted to an optical density of OD 0.5 at 595 nm before use.

### Antibodies and chemicals

Antibodies and chemicals used in the present study are listed as follows. Anti (α)-PRRSV-nsp1β PAb (rabbit polyclonal antibody) specific for PRRSV-2 nsp1β was generated at the Immunological Research Center, University of Illinois at Urbana-Champaign (Urbana, IL). α-N protein MAb (MR40; mouse monoclonal antibody) was obtained from E. Nelson (South Dakota State University, Brookings, SD). α-p65 Mab (mouse) (F-6, sc-8008) was purchased from Santa Cruz Biotechnologies Inc. (Santa Cruz, CA). Alexa-Flour 488-conjugated and Alexa-Flour 568-conjugated secondary antibodies were obtained from ThermoFisher (Rockford, IL). Human tumor necrosis factor-α (TNF-α) (8902) was purchased from Cell Signaling (Danvers, MA). DAPI (4′, 6′-diamidino-2-phenylindol) and Polyinosinic:polycytidylic [poly (I:C)] were obtained from Sigma (St. Louis, MO). Human and porcine recombinant IFN-β were purchased from Calbiochem (San Diego, CA), and for stimulation, 1000 unit/ml was added to cells for 6 h.

### Genes and plasmids

The genes for nsp1β and N were cloned from of PRRSV-2 strain VR2332 and inserted into the pXJ41 expression vector as described previously [33]. The mutant plasmids nsp1β-L126A and N-ΔNLS were constructed by PCR-based site-directed mutagenesis using specific primer pairs as follows; for nsp1β-L126A, forward 5’-TGCAGCCTCCGTTGTGCGTACTTGCCAGCGAC-3’, reverse 5’-GTCGCTGGCAAGTACGCACAACGGAGGCTGCA-3’; for N-ΔNLS, forward 5’- GGCAAGGGACCGGGAAATAAGAAGAAATCC-3’, reverse 5’- GGATTTCTTCTTATTTCCCGGTCCCTTGCC-3’. PCR-based mutagenesis was performed using the QuikChange II XL Site-Directed Mutagenesis kit (Agilent, Santa Clara, CA) according to the manufacturer’s instruction. The pIFN-β-luciferase reporter plasmid was kindly provided by Stephan Ludwig (Institute of Molecular Medicine, Heinrich Heine Universtät, Düsseldorf, Germany). The pNF-κB-luciferase reporter plasmid was purchased from Stratagene Inc (La Jolla, CA). The pRL-TK Renilla luciferase reporter plasmid was purchased from Promega (Madison, WI).

### Viral RNA isolation and RT-qPCR

Viral RNA from sera was extracted using the QIAamp Viral RNA mini kit according to the manufacturer’s instruction (QIAGEN). Viral RNA extraction from cells was carried out using TRIzol (Invitrogen). Briefly, one ml of TRIzol was added to cells, and the mixture was incubated for 5 min at room temperature (RT). Next, 0.2 ml of chloroform was added, and the mixture was shaken vigorously for 20 seconds and incubated for 3 minutes. After centrifugation at 12,000 rpm in a microcentrifuge for 15 minutes at 4°C, the aqueous phase was transferred to a fresh tube. Then, 0.6 ml of isopropyl alcohol was added, and the mixture was centrifuged again at 12,000 rpm for 10 minutes at 4°C. The RNA pellet was washed with 1 ml of 75% ethanol, air-dried, and dissolved in 30 μl of RNase-free water. The extracted RNA was stored at -80°C until use. To detect the viral sequences in the sera and PAMs, RT-PCR was carried out for the nsp1β and N genes using M-MLV reverse transcriptase (Invitrogen) and Taq DNA polymerase (Invitrogen) following the manufacturer’s instructions. Specific primers for the nsp1β and N gene amplifications were as follow; for nsp1β, forward 5’-TACAGGTTTATGAACGGGGTTG-3’, reverse 5’-GCGGGGAATAGTACTTGAGATG-3’; for N, forward 5’-GATAACCACGCATTTGTCGTC-3’, reverse 5’-TTGAACAAATTAAAACAAAAAGGTG-3’. The PCR products were sequenced at the Roy Carver Biotechnology Center of the UIUC.

### Construction of mutant PRRSV-2 infectious clones and generation of mutant viruses

The mutant virus PRRSV-2 vL126A was created by substituting the CTA codon for leucine at 126 to GCT for alanine in nsp1β using the P129 infectious clone [32]. vΔNLS was generated by deleting two lysine residues at 43 and 44 and substituting the asparagine at 49 with serine in N of the PRRSV infectious clone [75]. To create the vL126A/ΔNLS mutant, a long-range inverse PCR-based mutagenesis was performed using the vΔNLS infectious clone as a template, using the QuikChange II XL Site-Directed Mutagenesis Kit according to the manufacturer’s instructions (Agilent, Santa Clara, CA). To rescue mutant PRRSV-2 from infectious clones, MARC-145 cells were transfected with 2 μg of full-length DNA clone using Lipofectamine 2000 (Invitrogen, Carlsbad, CA). The culture supernatants were harvested at 6-day post-transfection and designated “passage-1.” The passage-1 virus was propagated in MARC-145 cells, and the 6-day harvest was designated “passage-2” virus. The “passage-3” and “passage-4” viruses were prepared in the same way as passage-2, and each passage was aliquoted and stored at -80°C until use. Viral infectivity was confirmed by the appearance of CPE and by IFA with PRRSV-2 nsp1β and N protein antibodies. For viremia, standard plaque assay was performed in MARC-145 cells. Briefly, cells were grown in 6-well plates as a monolayer and infected with 0.1 ml of 10-fold serial dilutions of sera collected at indicated days. Virus-infected cells were then overlaid with 0.8% agarose in DMEM and incubated at 37°C. Between 3 to 4 dpi, cell monolayers were stained with 5% crystal violet in 20% ethanol for 10 min and washed with water several times. The size of plaques was recorded by taking pictures. Each assay was conducted in duplicate and repeated twice. Viral titers were calculated and expressed as TCID_50_/ml. To confirm the successful generation of desired mutant viruses, viral genomic RNA was extracted using the QIAamp Viral RNA mini kit (QIAGEN, Hilden, Germany), and RT-PCR amplification was performed for the full nsp1β and N genes followed by sequencing to verify the desired mutations.

### JAK-inhibitor assay

To examine the replication efficiency of the double-mutant virus vL126A/ΔNLS, the JAK-inhibitor assay was conducted. (F), To determine relative expressions of ISGs, MARC-145 cells were seeded in 6-well plates and incubated with 1,000 units of human IFN-β for 2 h for activation of the JAK-STAT pathway. Then, the cells were treated with ruxolitinib at indicated concentrations (STEMCELL Technologies, Cambridge, MA). 24 h later, total cellular RNA was extracted for RT-qPCR for ISG15, ISG54, and ISG56. The relative fold changes of ISG transcripts were normalized to that of β-actin and statistically analyzed. ***, p<0.001. (G), MARC-145 cells were treated for 24 h at 1 μM concentration, followed by infection with vWT or vL126A/ΔNLS for 48 h. Culture supernatants were collected and titrated by tissue culture infectious dose 50 (TCID_50_) in 96-well plate using the Reed-Muench method. The experiment was conducted twice in duplicate each.

### Immunofluorescence analysis (IFA)

Virus-infected MARC-145 cells were grown on microscope coverslips and fixed with 4% paraformaldehyde in PBS for 30 min at RT. After three washes with PBS, cells were permeabilized with 0.1% Triton X-100 for 15 minutes at RT and then washed with PBS three more times. To block non-specific binding, cells on coverslip were incubated with 1% BSA in PBS for 30 min at RT. Next, primary antibody of 1:200 dilution in PBS containing 1% BSA was added to cells, and the cells were incubated for 2 h. After three washes with PBS, the cells were incubated with secondary antibody (1:200 dilution) for 1 h. The nuclei were stained with DAPI for 5 min at RT, and the coverslips were washed with PBS. Finally, coverslips were mounted onto microscope slides using Fluoromount-G mounting medium (Southern Biotech, Birmingham, AL), and signals were examined using the Nikon A1R confocal microscope.

### Dual luciferase reporter assay

To assess gene expressions driven by the IFN and NF-kB promoters, dual-luciferase reporter assays were conducted using the Dual-Glo Luciferase assay system (Promega) after DNA transfections using Lipofectamine 2000 (Invitrogen). Cells were grown in 12-well plates and transfected with 0.5 μg of viral gene, 0.5 μg of reporter plasmid, and 0.05 μg of pRL-TK in 1:1:0.1 ratio. To detect virus-induced reporter activities, cells were grown in 12-well plates and transfected with 0.5 μg of reporter plasmid and 0.05 μg of pRL-TK. At 6 hours post-transfection, the cells were infected with 1 MOI of respective virus. At 24 h post-transfection or post-infection with virus, 0.5 μg of poly(I:C) was transfected for 6 h for IFN induction. For induction of NF-κB, TNF-α (20 ng/ml) was added for 6 h, and cell lysates were prepared for luciferase assays. Luciferase activity was measured the Wallac 1420 VICTOR multi-label counter (Perkin Elmer, Waltham, MA). Renilla expression was used as an internal control for normalization, and the results were expressed as relative luciferase activity. Each assay was conducted in triplicate and repeated three times.

### Real time quantitative RT-PCR (RT-qPCR)

Total cellular RNA was extracted using the TRIzol reagent according to the manufacturer’s instruction (Invitrogen). RT-qPCR reactions were performed using the final volume of 25 μl containing 2.5 μl of cDNA, 12.5 μl of SYBR Green Master mix (Applied Biosystems), 2.5 μl of primer pairs [1.25 μl each of forward and reverse primers (10 mM)], and 7.5 μl of water in the ABI sequence detector system (ABI Prism 7000 and software; Applied Biosystems). The amplification parameters were 40 cycles of two steps each cycle comprised of heating to 95°C and 60°C. The primer sets used in assays were described in Table 2. The mRNA levels for target genes were calculated based on 2^-ΔΔCt^ method [79] and normalized using GAPDH as an internal control. Swine-specific oligonucleotides for RT-qPCR were synthesized at the Eurofins Genomics (Louisville, KY).

**Table 2:** Primer sets used for RT-qPCR in this study.

Name	Forward primer (5′ → 3′)	Reverse primer (5′ → 3′)
IL-1α	GTGCTCAAAACGAAGACGAACC	CATATTGCCATGCTTTTCCCAGAA
IL-1β	AACGTGCAGTCTATGGAGT	GAACACCACTTCTCTCTTCA
IL-6	CTGGCAGAAAACAACCTGAACC	TGATTCTCATCAAGCAGGTCTCC
IL-8	CCGTGTCAACATGACTTCCAA	GCCTCACAGAGAGCTGCAGAA
IL-10	CGGCGCTGTCATCAATTTCTG	CCCCTCTCTTGGAGCTTGCTA
IL-17	AATGCTGAAGGGAAGGAAGA	CCCACTGTCACCATCACTTT
GM-CSF	GCAGAACCTGCTTCTCCTG	GGCTCAGGGCTTCTTTGAT
RANTES	AGCATCAGCCTCCCCATATG	TTGCTGCTGGTGTAGAAATATTCC
TNFα	AACCTCAGATAAGCCCGTCG	ACCACCAGCTGGTTGTCTTT
IP-10	CTGCATCAAGATCAGTGACAGAC	TTGTGGCAATGATCTCAACAT
MCP1	GCAGCAAGTGTCCTAAAGAAGCA	GCTTGGGTTCTGCACAGATCT
MCP2	AAGACCAAAGCCGACAAGGA	TCATGGAATTCTGGACCCACTT
IFNβ	AGTGCATCCTCCAAATCGCT	GCTCATGGAAAGAGCTGTGGT
IFNγ	AATGGTAGCTCTGGGAAACTG	ACTTCTCTTCCGCTTTCTTAGG
β-actin	GTGCGGGACATCAAGGAGAAG	CAGGAAGGAGGGCTGGAAGAG

### Experimental infection of pigs

The animal infection and necropsies were conducted in the biosafety level 2 (BSL2) biocontainment facility of UIUC. A total of 39 piglets of 5-week-old, mixed-breed pigs were brought to the facility and pre-screened for the evidence of exposure to PRRSV, porcine circovirus, *S*. *suis*, and *Mycoplasma hyopneumoniae*. The animals were randomly allotted to 6 groups; 7 animals per infection group and 4 animals for mock infection group. The animals were housed in separate environmentally isolated pens within the facility. Throughout the study, pigs were handled according to the IACUC protocol and were fed an age-appropriate non-medicated diet. Feed and water were provided ad libitum, and pigs in the same group were allowed to freely mingle. On the day of infection, PRRSV-2 stocks were prepared to make 2×10^4^ TCID_50_/mL, and each pig was infected with a total of 4×10^4^ TCID_50_ by a single intranasal administration with 2 ml of virus. After infection, an aliquot of each inoculum was retitrated by TCID_50_ assay to confirm the dose of infection. The bacterial inoculation was conducted with 2 ml of 4×10^7^ colony-forming unit (CFU)/mL of *S*. *suis* through intranasal administration. Pigs were monitored daily for any symptoms of illness in consultation of the attending veterinarian. Blood samples were taken on 0, 4, 7, 9, 11, and 14 dpi for virus isolation and serology, and serum were aliquoted and stored immediately at −80°C until use. Pigs were weighed upon arrival at the site and on days collecting blood samples. The animals were euthanized on 14 dpi to end the study. Necropsies were performed by a board-certified veterinary anatomic pathologist that was blinded to the treatment of the animals. The extent of gross lung pathology was assessed by calculating a mean percentage value of the lung exhibiting gross visible pneumonia based on the percentage of each lobe exhibiting pneumonic changes. The overall percent of lung pathology for each lung was calculated using a standard scoring method [80,81]. Bronchoalveolar lavage (BAL) fluid was collected from the lungs, and PAMs were isolated from BAL as described elsewhere [82]. The lung tissue samples were taken and immersed in formalin for histopathological examination.

### Viremia and PRRSV-specific serum antibodies

Viremia was determined as a 50% tissue culture infectious dose (TCID_50_) in MARC-145 cells. Briefly, cells were grown in 96-well plates as a monolayer and infected with 0.05 ml of 10-fold serial dilutions of the serum collected at indicated days. At 3–4 dpi, cells were monitored for the development of cytopathic effects, and viral titers were calculated using the method of Muench and Reed [83]. Each sample was examined in triplicate. PRRSV-specific serum antibody was determined at the VDL of UIUC using the HerdCheck PRRS ELISA kit according to the manufacturer’s instruction (Westbrook, ME). The S/P ratio value of 0.4 was set as the cutoff for positive/ negative. S/P ratios greater than 0.4 were considered positive.

### Profiling porcine cytokine expressions

Porcine cytokine expressions in the lung lavages were determined using the Multiplex for Luminex Immunoassays according to the manufacture instructions (PCYTMG-23K-13PX, Millipore Sigma, St. Louis, MO). Quality controls and standard controls were first established for the porcine cytokine/chemokine-antibody immobilized magnetic bead panel. On day 1, 200 μl of assay buffer was added to each well of the plate and mixed on a shaker for 10 min at RT. The buffer was discarded and 25 μl each of the standard buffer, assay buffers, and matrix solution was added to appropriate wells. An equal volume of samples was added, and the plate was incubated overnight in the dark at 4 C in a plate shaker. On day 2, the plate was screwed tightly on the handheld magnet and the content was discarded. The plate was washed three times in the same way, and 50 μl of detection antibodies was added to each well for 2 h incubation, followed by adding 50 μL of Streptavidin-Phycoerythrin to each well. The plate was incubated for 30 min at RT and washed three times with washing buffer. After the final wash, the beads were resuspended with 100 μl of washing buffer for 5 min. The plate was read in the multiplex analyzer (Luminex 200 System, DiaSorin; Stillwater, MN), and the data were analyzed using the accompanied software (Luminex SD xMAP Technology LX100/200).

### Statistical analysis

Statistical significance was determined by a two-tailed Student’s t-test. Data analyses were performed using GraphPad Prism version 9.00 (San Diego California USA).
